# The Relationship between the Structure and Properties of Amino Acid Ionic Liquids

**DOI:** 10.3390/molecules24183252

**Published:** 2019-09-06

**Authors:** Paula Ossowicz, Joanna Klebeko, Barbara Roman, Ewa Janus, Zbigniew Rozwadowski

**Affiliations:** 1 Department of Organic Chemical Technology, Faculty of Chemical Technology and Engineering, West Pomeranian University of Technology, Piastów Ave. 42, 71-065 Szczecin, Poland (J.K.) (B.R.) (E.J.); 2 Department of Inorganic and Analytical Chemistry, Faculty of Chemical Technology and Engineering, West Pomeranian University of Technology, Piastów Ave. 42, 71-065 Szczecin, Poland

**Keywords:** amino acid ionic liquids, physical properties, dissolution of cellulose

## Abstract

Ionic liquids based on different l-amino acids (glycine, l-valine, l-leucine, l-isoleucine, l-histidine, l-methionine, l-tyrosine, l-tryptophan, l-arginine, and l-threonine) and different cations (tetrabutylammonium (TBA), tributylmethylammonium (tBMA), didecyldimethylammonium (DDA), (2-hydroxyethyl)trimethylammonium (choline) (Chol), alkyl(C_12_-C_14_) dimethylbenzylammonium (benzalkonium) (BA), dodecyltrimethylammonium (DDTMA), hexadecyltrimethylammonium (HDTMA), octadecyltrimethylammonium (ODTMA) and 1-ethyl-3-methylimidazolium (EMIM)) have been synthesized and characterized by NMR and FTIR. Viscosity, specific rotation, surface activity, thermal stability (TG), and phase transformations (DSC) have been determined and compared with available data. Furthermore, benzalkonium, didecyldimethylammonium, dodecyltrimethylammonium, hexadecyltrimethylammonium, and octadecyltrimethylammonium amino acid ionic liquids have been shown to exhibit surface activity. The dissolution of cellulose in amino acid ionic liquids (AAILs) composed of various cations was also investigated. Cellulose was only dissolved in EMIM salts of amino acids. In particular, the influence of the cation type on selected physicochemical and spectroscopic properties were discussed. The article is a mini review on amino acid ionic liquids.

## 1. Introduction

Ionic liquids have been described as “designer solvents”, and this means that their properties can be adjusted to the requirements of the process. The most important properties are negligibly low vapor pressure, high thermal stability, ability of dissolving various materials, and high ionic conductivity. Some properties, such as melting point, viscosity, density, and hydrophobicity, can be varied by simple changes of the structure of ions. Ionic liquids are used as solvents in many reactions and as electrolytes [1,2,3,4,5]. Several years of investigation on the synthesis and application of ionic liquids have shown that they can be the perfect solution for various technological problems. Their usefulness is determined by their structure, which can be modified in a wide range. Environmental aspects as well as “green chemistry” rules have meant that attention has focused on the use of renewable raw materials in the design and synthesis of ionic liquids. Thus, in recent years, ionic liquids have been prepared from amino acids, sugars, and terpenes [6,7,8,9,10,11,12]. An additional advantage of these raw materials is their optical activity, which can expand the potential applications of ionic liquids prepared based on them to bioprocesses, biotechnology, and asymmetric synthesis.

Amino acids are specific raw materials in the synthesis of ionic liquids. They can be the source of both cations and anions. They are chiral and have two chemically active centers suitable for modification [12,13,14]. Amino acid ionic liquids (AAILs) have a high hydrogen bonding ability useful for dissolution of biomaterials such as DNA, cellulose, and other carbohydrates [15]. The strong hydrogen bonding ability of AAILs as compared to conventional ionic liquids makes AAILs more demanding as chiral solvents and reagents for dissolution and stabilization of biomolecules (such as carbohydrates, cellulose, nucleic acids, etc.) in medicinal, synthetic, and pharmaceutical chemistry [16,17]. In industrial and pharmaceutical chemistry, ionic liquids based on amino acids have many different applications such as an intermediate in the synthesis of peptides, chiral solvents, and absorbents for acid gases [18]. Moreover, they have proved to be effective catalysts for many reactions, i.e., asymmetric aldol condensation between aldehydes and ketones in the presence of water [19], Diels–Alder reaction, and asymmetric Michael addition [20,21,22].

Due to their distinctive properties such as tunable hydrophobicity, hydrogen bonding ability (proton-donating/accepting properties), unique acid-base characteristic amino acid ionic liquids (AAILs) have tremendous potential in the field of enzymatic reactions, protein chemistry, and as templates for synthetic study.

Several amino acid ionic liquids, such as tetrabutylphosphonium [23], tetrabutylammonium [24], cholinium [25], 1-butyl-3-methylimidazolium [26] and 1-ethyl-3-methylimidazolium [27,28] salts of 20 natural l-amino acids are known. However, not all of their properties have been previously reported and compared.

In this study, we present a comparison of the physicochemical and spectroscopic properties of several amino acid ionic liquids composed of different cations—tetrabutylammonium (TBA), tributylmethylammonium (tBMA), didecyldimethylammonium (DDA), (2-hydroxyethyl)trimethylammonium (choline) (Chol), alkyl(C12-C14) dimethylbenzylammonium (benzalkonium) (BA), dodecyltrimethylammonium (DDTMA), hexadecyltrimethylammonium (HDTMA), octadecyltrimethylammonium (ODTMA), and 1-ethyl-3-methylimidazolium (EMIM). In addition to various cations, different amino acid anions have also been selected to compare the impact of the hydroxyl group (l-Thr), thioether functional group (l-Met), phenol group (l-Tyr), guanidino group (l-Arg), an imidazole (l-His) or indole ring (l-Trp), and the length and structure of the alkyl chain at the chiral carbon of amino acid (Gly, l-Val, l-Leu, l-Ile). For the studied salts, the properties which are important from a practical point of view, such as solubility, thermal stability, optical activity, and surface activity, as well as solubility of cellulose, were tested and compared.

TBA salts of these amino acids have been previously used by us to synthesize amino acid ionic liquid-supported Schiff bases, which have been shown to be capable of forming intramolecular hydrogen bonds. It was found that the balance between the NH and OH form in these ionic liquids depends on the amino acid [29].

## 2. Results and Discussion

**Scheme 1:**
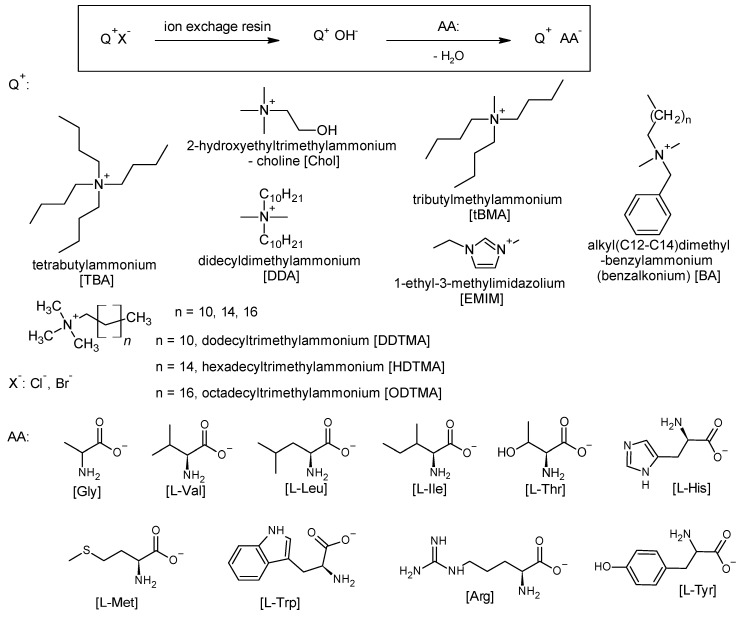
Synthesis method of AAILs.

Organic salts of amino acids were prepared by proton exchange in the amino acid carboxyl group by the organic cation, according to the previously described procedure (Scheme 1) [1,30].

The synthesis of amino acid ionic liquids, without tetrabutylammonium ionic liquids, consisted of two stages. In the first step, the halide anion in organic halides was exchanged for a hydroxide anion on the ion exchange resin.

In the second step, amino acid (1.2 equivalents) was dissolved in the aqueous solution of organic hydroxide (one equivalent). After the procedure described in Section 3.2, the product was dried in a vacuum oven at 60 °C to a water content below 800 ppm (at least 24 h). All products were obtained with high yields (85–99%) and high purity. All AAILs were identified by NMR spectroscopy. The complete assignments of ^1^H NMR and ^13^C NMR were presented only for new tBMA, DDTMA, HDTMA, and ODTMA salts of amino acids because TBA, DDA, BA, Chol, and EMIM salts have been previously described [1,23,24,25,30,31,32,33,34]. However, the spectroscopic and physicochemical data have never been compared to each other.

### 2.1. Spectroscopic Properties 

The ^1^H and ^13^C chemical shifts for α-positions in amino acids and ^13^C chemical shifts for the carboxyl group in DMSO-d_6_ are collected in Table 1.

The chemical shift values for H-α of amino acid anions were similar for all salts. Owing to the nature of the cation, the highest chemical shift values (δ2.69–3.57 ppm) were found for benzalkonium ionic liquids. In general, considering the kind of the anion, the lowest chemical shift values were exhibited by aliphatic amino acids (l-Val or l-Leu) and the highest by aromatic and/or heterocyclic amino acids (l-His, l-Trp, l-Tyr).

The largest differences in chemical shift value in ^13^C spectra were observed for carbon C=O (C-2′). The values were in the range of 172.2 up to 185.8 ppm. These values confirmed the ionic structure of compounds [29,36,37,38]. The difference between the values observed for benzalkonium and other ionic liquids as about 2.4–9.3 ppm. The differences in chemical shifts for carbon C=O (C-2′) are due to the presence of the aromatic ring in the benzalkonium cation. It seems that the aromatic ring of the benzalkonium cation is in the vicinity of this anion fragment and is positioned such that the carbon C=O and the proton H-α are under its influence. No apparent effect on carbon C-α offset suggests that it is outside its influence. A similar, but significantly less pronounced effect can be seen in EMIM derivatives. These effects are also caused by the length of the alkyl substituent, because ODTMA, HDTMA, and DDTMA show similar effects. The influence of the hydroxyl group on the choline derivative is definitely lower but there must be some interaction between the OH group and the COO^−^ group.

**Figure 1:**
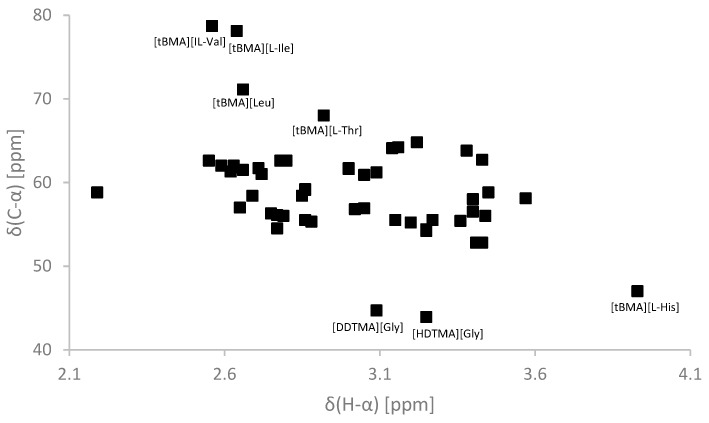
Plot of C-α chemical shifts vs. H-α chemical shifts (ppm).

No relationship between H-α and C-α chemical shifts was observed (Figure 1). C-α is relatively insensitive to the type of cation and amino acid anion. Interestingly, all deviations refer to derivatives with an asymmetric cation. The largest deviations were found for tBMA, DDTMA, and HDTMA, respectively. The biggest influence on the chemical shifts carbon C-α was observed for tBMA cations. Chemical shifts in tBMA derivatives are significantly higher than in other derivatives. Perhaps this is related to some unsymmetrical interaction between longer cation fragments and carbon.

FTIR spectra of respective amino acid ionic liquids are compared and collected in Table 2 (most distinctive IR bands). The data from the Table 2, i.e., the influence of the position of the characteristic bands depending on the type of cation, are presented to show the impact of the interaction between the cation and anion in the ionic liquid.

The broad band ca. 3000–3600 cm^−1^ was assigned to *v*N–H and *v*O–H. The strong band at ca. 2960 cm^−1^ was assigned to *v*C–H The strong bands at ca. 1570 and 1390 cm^−1^ were assigned to *v(*COO^−^)_sym._ and *v(*COO^−^)_as_, respectively [29,35,39]. The differences of the IR bands for the derivatives of various amino acids and the same cations were negligible. 

### 2.2. Physicochemical Properties 

**Figure 2:**
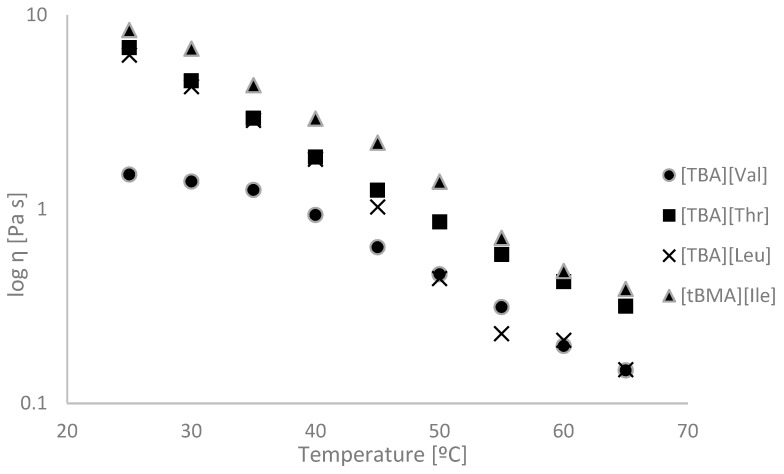
Variation in the viscosities of the tetrabutylammonium amino acid ionic liquids (AAILs) with temperature.

**Figure 3:**
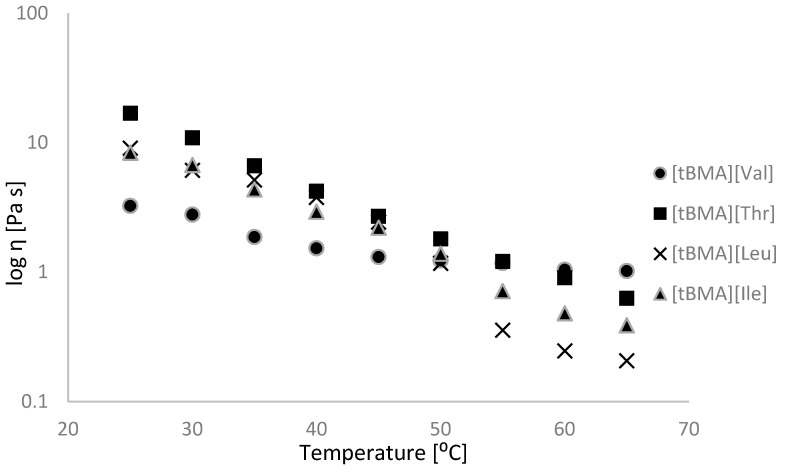
Variation in the viscosities of the tributylmethylammonium AAILs with temperature.

**Figure 4:**
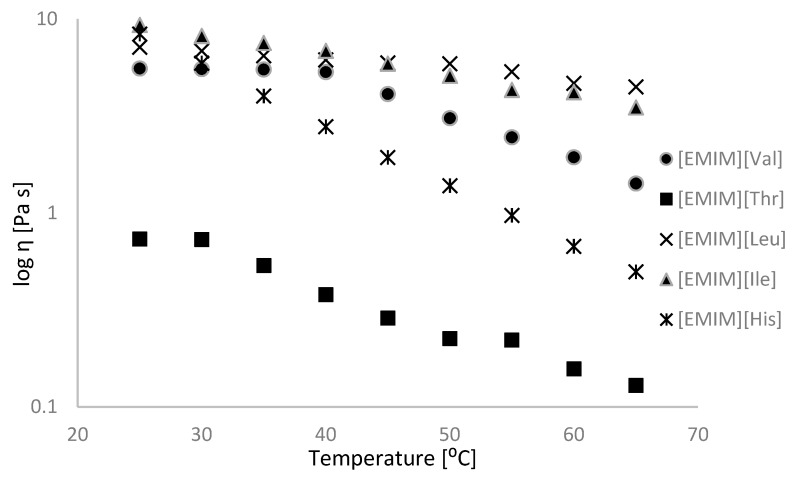
Variation in the viscosities of the 1-ethyl-3-methylimidazolium AAILs with temperature.

**Figure 5:**
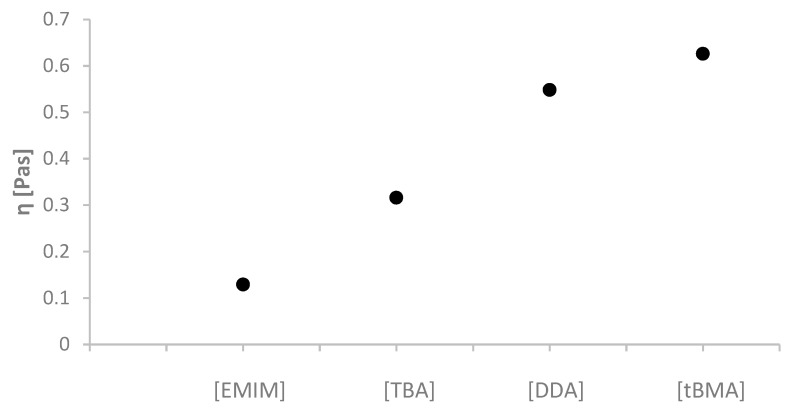
Effect of cation type on the viscosity, η, for threonine-based ionic liquids.

Most of the prepared organic salts of amino acids were colourless or slightly yellow liquids at room temperature (Table 3). Salts of histidine—[TBA][l-His], [tBMA][l-His], and tryptophan [TBA][Trp] were exceptions because they melt at 128.5 °C, 58.9 °C, and 129.6 °C, respectively. The viscosities of the amino acids were recorded at different temperatures and are summarized in Figure 2, Figure 3, Figure 4 and Figure 5. The viscosities at 65 °C are presented in Table 3. The viscosity of the amino acid ionic liquids at 25 °C ranged from 330 to 16,856 mPa∙s. TBA salts have the lowest viscosity among the studied compounds. The viscosity decreased significantly with increasing temperature (Figure 2, Figure 3 and Figure 4). As indicated in Figure 2, Figure 3 and Figure 4, the viscosity of the ILs is sensitive to temperature, e.g., the viscosity sharply changes when the ILs are in lower temperatures, the viscosity of the ILs with high viscosity values is especially sensitive to temperature. Such sensitivity of IL viscosity to temperature has been indicated in other studies [40,41,42,43,44]. The viscosities are largely dependent on the nature of the cation. The effect of cation type on AAIL viscosity for the threonine anion is reported in Figure 5. The viscosity values shows the salts with imidazolium cations are less viscous than the quaternary ammonium-based ILs. These trends are known and typical of other ILs with different anions [45]. Asymmetric N-substituted imidazolium ionic liquids owe their low viscosity to the synergistic effects of charge delocalization and planarity leads. The viscosity of tBMA and DDA are remarkably larger than that for other considered ILs, as it may be expected from the asymmetry of the alkyl substituents. There is also a significant difference in viscosity of the liquid with various anions. H-bonding is also a factor affecting the viscosities of ILs. Compounds with fewer hydrogen bonds have a lower viscosity [46]. In our case, fewer hydrogen bonds in ammonium AAILs do not decrease their viscosity relative to the imidazolium AAILs, which is associated with conjugated cation structure. The positive charge of the imidazolium cation is well distributed, which remarkably weakens the Coulomb interactions among ions. As a result, the viscosity of imidazolium AAILs are lower than that of quaternary ammonium AAILs. The asymmetry of the cation does have a significant impact on the viscosity. It is generally known that ionic liquids with asymmetric cations have a lower viscosity than those with symmetric cations. However, after a detailed analysis of literary data, it was shown that it depends on the type of substituent. For example, the viscosity of [N_2222_][l-Ala] is 81 mPa∙s at 25 °C, while for [N_2224_][l-Ala] the viscosity is merely 29 mPa∙s, and for [N_2221_][l-Ala] it is 84 mPa∙s [47]. The molecular size and asymmetry of the anions also apparently influence the viscosity of AAILs. It is seen in Figure 2 and Figure 3 that the viscosities of TBA and tBMA amino acids generally decrease in the order of Thr > Leu > Ile > Val. The highest viscosity was observed in liquids with Thr anions, which is associated with the presence of an additional polar –OH group in the structure. From Figure 1, a rapid decrease in the viscosities is found in the AAILs as the temperature increases. The influence of temperature on the viscosity is very significant at lower temperatures. At a higher temperature of 65 °C, the viscosity of [TBA][l-Val] is 148 mPa∙s and that of [TBA][l-Leu] is 149 mPa∙s, which are lower than that of [TBA][l-Ile] (176 mPa∙s) and [TBA][l-Thr] (316 mPa∙s). The strength of the momentum transfer of threonine ionioc liquid is more temperature-dependent than other tetrabutylammonium ILs. The viscosity variation of [TBA][Thr] and [tBMA][Thr] indicates that the interaction forces between the cation and anion are sensitive to temperature.

**Figure 6:**
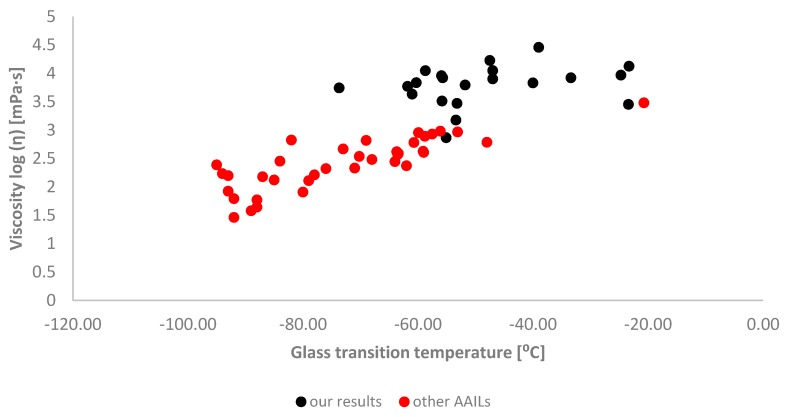
Relation between viscosity (25 °C) and glass transition temperature (T_g_) of amino acid ionic liquids.

Figure 6 shows the relation between T_g_ and viscosity (at 25 °C) for different AAILs. All AAIls have a linear relationship between viscosity and T_g_. Thus, for these ionic liquids, the side-chain structure did not affect the general relationship between T_g_ and viscosity. 

**Figure 7:**
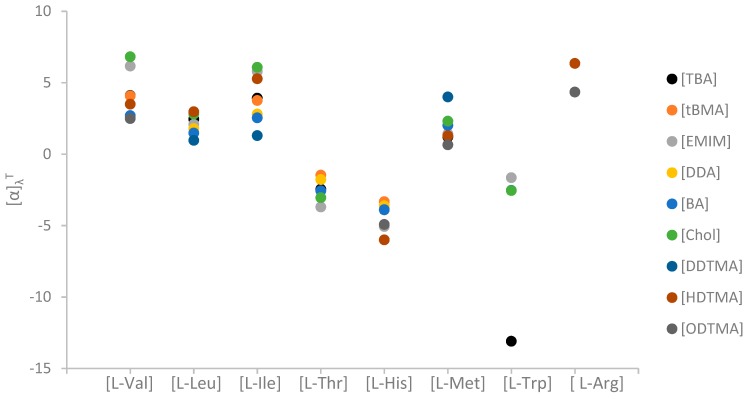
Plot of specific rotation ([α]) vs. amino acids.

**Figure 8:**
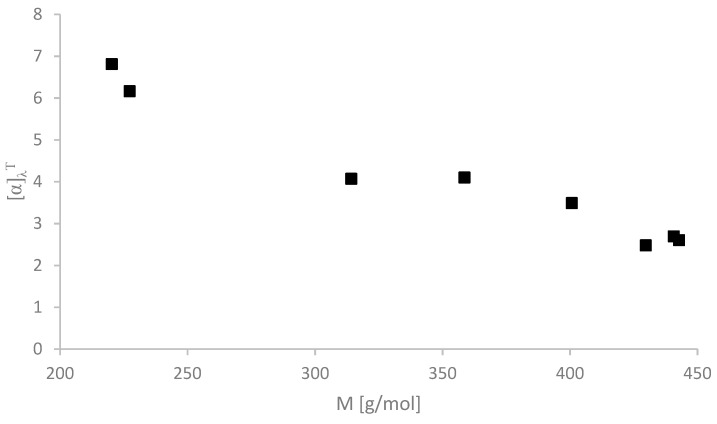
Plot of specific rotation ([α]) vs. molar mass for tetrabutylamonnium ionic liquids.

All obtained AAILs are chiral with specific rotation listed in Table 3. Specific rotation was similar for most of the salts (with different cation) of the same amino acid ([α]_λ_^T^, Figure 7). Some trends were observed between specific rotation and molar mass (Figure 8). Such relationships suggest that [α] changes with the size of molecules (especially the size of the cation).Given that amino acids have the ability to rotate polarized light, it is reasonable that an increase in the molar ratio of the cationic part decreases the absolute value of the rotation.

Differential scanning calorimetry (DSC) showed the glass transition for the studied ionic liquids (T_g_, Table 3). It has been observed that the glass transition depends on the cation structure. Ionic liquids with a symmetrical tetrabutylammonium cation showed higher glass transition temperature in comparison to those of tributylmethylammonium and 1-ethyl-3-methylimidazolium salts. Furthermore, the highest temperature of glass transition was found for threonine and histidine derivatives.

**Figure 9:**
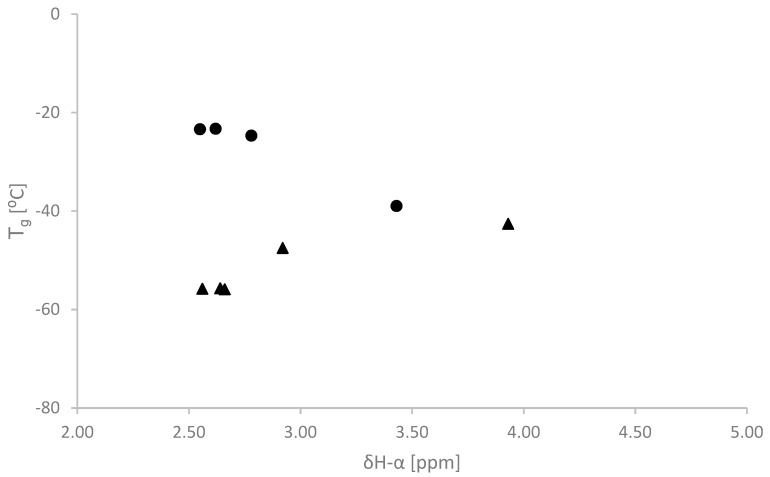
Plot of glass transition temperatures (T_g_) vs. H-α chemical shifts for didecyldimethylammonium (circles) and tributylmethylammonium (triangles) ionic liquids.

Some trends in the change of glass transition temperatures T_g_ with H-α chemical shift were observed, which are plotted for tBMA and DDA salts in Figure 9. These relationships may suggest that T_g_ depends on the structure of the amino acid anion. For amino acid ionic liquids with didecyldimethylammonium cation, along with a decrease in the glass transition temperature, there is increased H-α chemical shift (Figure 9, circle marked), which is contrary to other salts studied (such as tributylmethylammonium, Figure 9, triangles).

**Figure 10:**
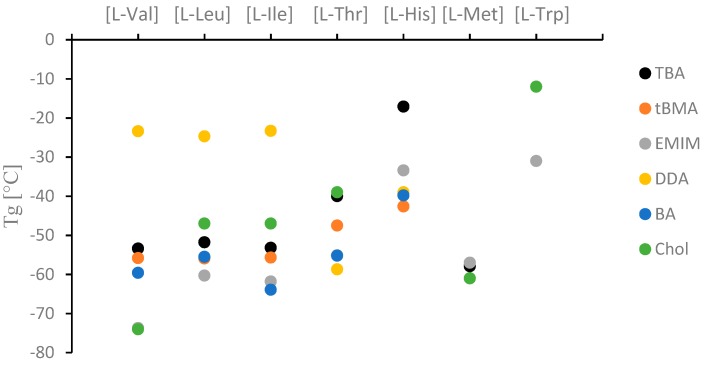
Plot of glass transition temperatures (T_g_) vs. amino acids.

The glass transition temperature dependence on the type of amino acid for the various tested cations is presented in Figure 10. Generally, it can be seen that the glass transition temperature is dependent on the structure of the cation of the ionic liquid. Hydrogen bonding, van der Waals interactions, and the size of the amino acid anion caused increases in thermal stability [1,15]. It is also noted that the glass transition temperature generally increases with increasing molecular weight of the amino acid.

Decomposition temperatures corresponding to 5% weight loss (T_d_^5%^) were in the range of 115.1 to 315.0 °C. 1-Ethyl-3-methylimidazolium salts of amino acids started decomposition at about 200 °C (except [EMIM][l-Thr] with T_d_^5%^ at 169.11 °C) and were the most stable among all prepared salts. AAILs with ammonium cations were less stable. They decomposed at about 120 °C.

**Figure 11:**
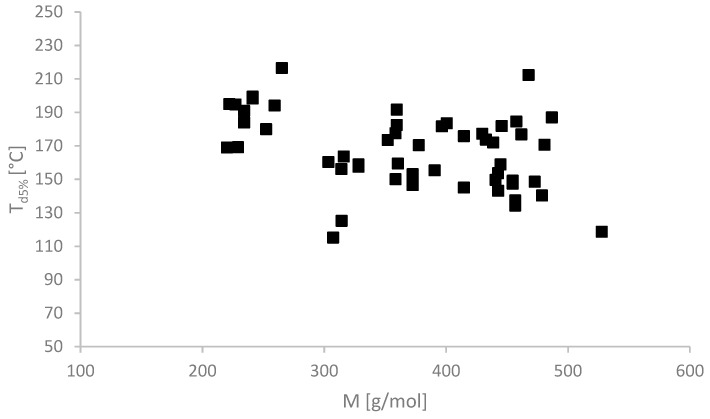
Plot of 5% (T_d_^5%^) weight loss temperature vs. molar mass for the compound studied.

**Figure 12:**
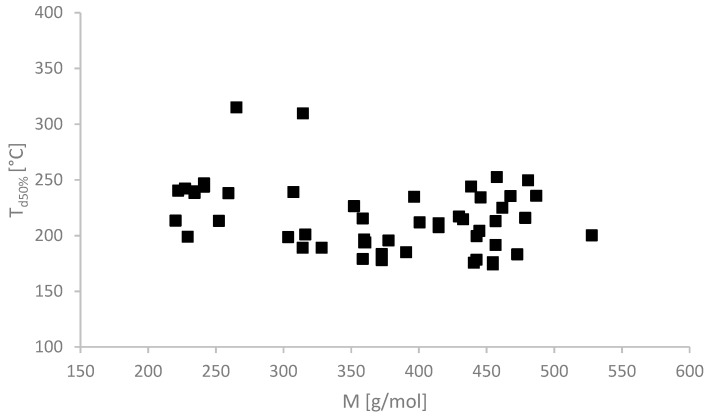
Plot of (T_d_^50%^) weight loss temperature vs. molar mass for the compound studied.

Small dependencies were observed between decomposition temperatures corresponding to 5% weight loss (Figure 11) or 50% weight loss (Figure 12) of the molecular weight. This relation indicates that the decomposition temperature depends on the structure of the compound studied and changes in the size of the molecules. This implies that the lower the molecular weight, the higher the stability of the compound.

**Figure 13:**
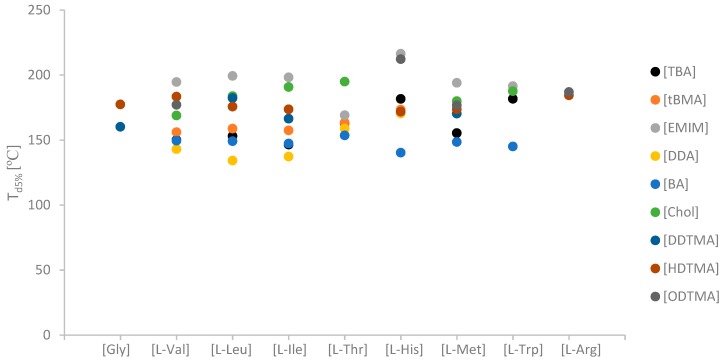
Plot of 5% (T_d_^5%^) weight loss temperature vs. amino acids.

**Figure 14:**
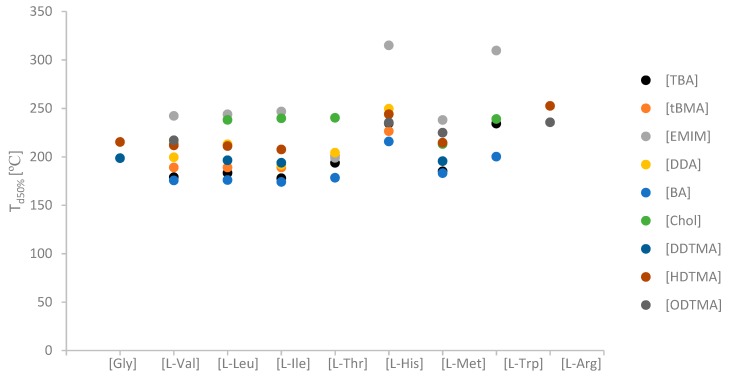
Plot of 50% (T_d_^50%^) weight loss temperature vs. amino acids.

Furthermore, the relationship decomposition temperatures corresponding to 5% weight loss and 50% weight loss and the type of amino acid for the various tested cations are shown in Figure 13 and Figure 14. This relation indicates that the decomposition temperature depends on the structure of the compound studied and changes in the size of the molecules. This implies that the lower the molecular weight, the higher the stability of the compound.

The miscibility with conventional organic solvents and water was investigated and is summarized in Table 4. The solvents were ranked by decreasing polarity index [48].

All AAILs were immiscible with nonpolar solvents such as benzene, diethyl ether, and *n*-hexane and were miscible with highly polar solvents such as water, mostly dissolved in acetone and ethanol. Most of them were miscible with ethyl acetate and chloroform. Exceptions were DDTMA, HDTMA, and ODTMA salts, which were immiscible, and [tBMA][l-His], [TBA][l-Trp], [Chol][l-Trp], which were only partly miscible with these solvents, and [DDA][l-His] and [TBA][l-Thr] were partly miscible with chloroform, and [TBA][l-His] was partly miscible with ethyl acetate.

Amino acid ionic liquids composed of DDA, BA, DDTMA, HDTMA, or ODTMA cations showed surface activity in aqueous solution (Table 5). The surface tension reached a minimum value between 32.3 and 33.8 mN·m^−1^ for benzalkonium amino acid salts, between 27.9 and 29.4 mN·m^−1^ for didecydimethylammonium salts, and between 40.1 and 42 mN·m^−1^ for dodecyltrimethylammonium, hexadecyltrimethylammonium, and octadecylammonium salts at critical micellar concentration (CMC). The CMC values were in the range from 0.25 mmol·L^−1^ to 0.34 mmol·L^−1^ for benzalkonium, from 0.50 mmol·L^−1^ to 0.61 mmol·L^−1^ for octadecyltrimethylammonium, from 0.60 mmol·L^−1^ to 0.87 mmol·L^−1^ for didecyldimethylammonium salts, from 0.74 mmol·L-1 to 1.09 mmol·L-1 for hexadecyltrimethylammonium, and from 11.81 mmol·L^−1^ to 12.26 mmol·L^−1^ for dodecyltrimethylammonium depending on amino acid anion. The area occupied per molecule at interphase *A_min_* of [ODTMA][l-Met] (6.635·10^19^ m^2^) was higher than for other salts, indicating that the molecules of AAILs containing ODTMA were more loosely packed at the water–air interface. The opposite situation was observed for [BA][l-Met], where the area per molecule was the smallest. The area per molecule *A_min_* was higher for didecyldimethylammonium salts than for other salts of the same amino acid.

The obtained ionic liquids were also tested as solvents of cellulose. We found that among the prepared AAILs, 1-ethyl-3-methylimidazolium salts of amino acids dissolved cellulose (Table 6). It is known from the literature that commercially available 1-ethyl-3-methylimidazolium chloride [EMIM][Cl] dissolves cellulose, displaying 31.4 mg g^−1^ solubility at 85 °C. However, [EMIM][Cl] is a solid with a melting point of 85 °C and requires heating above this temperature to dissolve the cellulose [49]. In comparison with the 1-ethyl-3-methylimidazolium chloride, EMIM salts of AA require a lower temperature. Solubility of cellulose at 60 °C in [EMIM][AA] (Table 6) is between 13.7 mg g^−1^ for [EMIM][l-Ile] to 43.2 mg g^−1^ for [EMIM][l-Thr]. For comparison, the recently published results have shown much lower solubility of cellulose in choline salts of AA; the concentration of cellulose was lower than 5 mg g^−1^ (Table 6). It is generally recognized that, in order to dissolve cellulose, its great number of inter- and intramolecular hydrogen bonds must be disrupted. Hydrogen bonding properties are important in solvents for the dissolution of cellulose. For this reason, EMIM-based ionic liquids have greater cellulose dissolution capacity.

## 3. Materials and Methods

### 3.1. Materials

Tetrabutylammonium hydroxide [TBA]OH (40% solution in H_2_O), 1-ethyl-3-methylimidazolium chloride [EMIM]Cl (≥95%), (2-hydroxyethyl)trimethylammonium chloride–choline chloride [Chol]Cl (≥97%), alkyl(C_12_-C_14_) dimethylbenzylammonium chloride–benzalkonium chloride [BA]Cl (≥95%), and microcrystalline cellulose (Avicel) were purchased from Fluka (Buchs, Switzerland). Tributylmethylammonium chloride [tBMA]Cl (75% solution in H_2_O) and didecyldimethylammonium chloride [DDA]Cl (~50% (2-propanol/water 2:3)), l-isoleucine (l-Ile) were purchased from MERCK (Darmstadt, Germany). Dodecyltrimethylammonium chloride [DDTMA]Cl (>97.0%) and octadecyltrimethylammonium chloride [ODTMA]Cl (>98.0%) were purchased from TCI (Nihonbashi-honcho, Chuo-ku, Japan). Hexadecyltrimethylammonium bromide [HDTMA]Br (>99.0%) was purchased from Acros Organics (Geel, Belgium). Glycine (Gly) (99.0%), l-histidine (l-His) (≥99.0%), l-leucine (l-Lys) (≥98.0%), l-tryptophan (l-Trp) (≥98.0%), l-tyrosine (l-Tyr) (≥98.0%) were provided by Sigma-Aldrich (Saint Louis, MO, USA). l-methionine (l-Met) (≥99.0%), l-arginine (≥99.0%), l-threonine (l-Thr) (≥99.0%) and l-valine (l-Val) (≥99.0%) were purchased from ROTH (Karlsruhe, Germany). Acetonitrile (99.5%) was provided by POCh (Gliwice, Poland). Ethanol (99.8%) was purchased from CHEMPUR (Piekary Śląskie, Poland). Dowex Monosphere 550 A UPW (OH form) resin was provided by Sigma-Aldrich (Saint Louis, MO, USA).

### 3.2. Preparation of Amino Acid Ionic Liquids

All AAILs were prepared by the reaction of amino acid and organic hydroxide. Organic hydroxides were commercial products, such as [TBA]OH, or obtained by ion exchange on the ion exchange resin from their respective organic halides ([BA]Cl, [Chol]Cl, [DDA]Cl, [EMIM]Cl, [tBMA]Cl), [DDTMA]Cl, [HDTMA]Br, [ODTMA]Cl). For this purpose, an aqueous solution of organic halide was placed on the top of glass column filled with ion exchange resin and then organic hydroxide was slowly eluted with deionized water. In collected eluates, the content of chloride anions was checked with AgNO_3_ and eluates without chloride anions were used in the second synthesis step.

Amino acid (1.2 equivalents) was dissolved in the aqueous solution of the corresponding organic hydroxide (1 equivalent). Glycine, l-valine, l-leucine, l-isoleucine, l-histidine, l-methionine, l-tyrosine, l-tryptophan, l-arginine, and l-threonine were chosen as amino acids. The mixture was stirred at room temperature for 24 h. Then, water was evaporated at 60 °C under vacuum by using a rotary evaporator. The excess amino acid was precipitated from residue with absolute ethanol and filtered. Then, ethanol was distilled off under vacuum from filtrate. The product was dried in vacuo for 24 h at 60 °C [29,30].

### 3.3. General Analytical Procedures

#### 3.3.1. Structural Characterization of Obtained Amino Acid Ionic Liquids

The prepared AAILs were identified by ^1^H NMR and ^13^C NMR. The ^1^H and ^13^C NMR spectra were recorded in DMSO-d_6_ or D_2_O on a BRUKER DPX-400 Avance III HD spectrometer (Billerica, MA, USA) operating at 400.13 MHz (^1^H) and 100.62 MHz (^13^C). TMS was used as an internal standard. 

FTIR spectra (KBr) were recorder in the 4000–400 cm^−1^ range, employing a Thermo Fisher Scientific Nicolet 380 FT-IR Spectrometer (Waltham, MA, USA) as thin film on KBr tablets.

#### 3.3.2. Water Content

The water content in AAILs was determined by coulometric Karl–Fischer titration, using a Metrohm 831 KF Coulometer (Herisau, Switzerland).

#### 3.3.3. Specific Rotation

The specific rotation [α]_D_^20^ measurements were taken with AUTOPOL IV Polarimeter from Rudolph Research Analytical (Hackettstown, NJ, USA) for aqueous solutions of AAILS (c = 0.01 g cm^−3^). Measurement was performed at 20 °C.

The physicochemical properties such as viscosity, thermal stability (TG), and phase transformations (DSC) were also determined.

#### 3.3.4. Viscosity

The viscosities of AAILs were measured at different temperatures (from 25 °C to 65 °C) using ARES (Advanced Rheometric Expansion System) Rheometric Scientific Rheometer (Piscataway, NJ, USA). During the viscosity measurement, the samples were blown with compressed dried synthetic air. This procedure protects against humidity absorption into ionic liquids.

#### 3.3.5. Thermogravimetric Analysis 

Thermogravimetric analysis was performed on thermomicrobalance TG 209 F1 Libra^®^ from NETZSCH (Selb, Germany), in Al_2_O_3_ crucible. Samples between 5–10 mg were heated from 25 °C to 1000 °C with a heating rate of 10 °C min^−1^, under air atmosphere (flow rate: air—25 cm^3^ min^−1^, nitrogen (as protective gas)—10 cm^3^ min^−1^). Temperatures corresponded to 5% mass loss (T_d_^5%^) and 50% mass loss (T_d_^50%^).

#### 3.3.6. Phase Transformations (DSC)

Phase transformation temperatures (glass transition temperature and melting point) were measured using a DSC analyzer (model Q-100, TA Instruments (New Castle, DE, USA)). Measurements were performed within the temperature range of −75 °C to 200 °C, in nitrogen atmosphere. The heating rate was 10 °C min^−1^. The sample was loaded on an aluminum pan sealed with a pinhole cap. Phase transition temperatures were determined according to ISO 11357-1:2009(E) using the midpoint temperature. Indium and mercury were used as standards to calibrate the temperature. Heat calibration used indium.

#### 3.3.7. Measurement of Surface Tension

Some of the prepared ionic liquids (with didecyldimethylammonium, benzalkonium, dodecyltrimethylammonium, hexadecyltrimethylammonium, and octadecyltrimethylammonium cation) exhibit surface activity. The critical micelle concentration and surface tension were measured by digital tensiometer (Krüss, K10ST, Hamburg, Germany) using the ring method at 25 °C. The values of the critical micelle concentration (CMC) and the surface tension at the CMC (γ_CMC_) were determined from the intersection of two straight lines drawn in low and high concentration regions in surface tension curves using a linear regression analysis method. Surface excess concentrations (Γ_max_) were calculated from the slope of linear portion of the γ–log c plots using the Gibbs isotherm:

(1) $$\Gamma_{max}=-\frac{1}{RT}\cdot \frac{d\gamma }{d(\ln c)}$$

where:

Γ_max_—surface excess concentration at the saturated interface, mol·m^−2^,
R—gas constant, 8.3144598(48) J·mol^−1^·K^−1^,
T—absolute temperature, K,
c—concentration of the salt, mol·dm^−3^.

From Γ_max_, the minimum surface occupied by a molecule at the interface A_min_ can be calculated from the equation:

(2) $$A_{min}=\;\frac{1}{\Gamma_{max}N_{A}}$$

where:

N_A_—the Avogadro number.

#### 3.3.8. Cellulose Solubility

To determine cellulose solubility, 0.1–0.5 mg sample was added to a glass vial containing 0.5 g IL at 60 °C under N_2_ with stirring and was visually checked to determine whether it was soluble. If the solution was clear, the next portion of cellulose was added. The solubility was calculated when the solution remained heterogeneous for 24 h.

The cellulose concentration can be calculated by the formula:

(3) $$C_{cel}=\;\frac{m_{cel}}{m_{cel}+m_{cj}}\cdot 100\%$$

where:

C_cel_—cellulose concentration [%],
m_cel_—weight of cellulose dissolved in the ionic liquid [g],
m_cj_—weight of ionic liquids [g].

## 4. Conclusions 

Amino acid ionic liquids were synthesized and investigated. Their NMR and FTIR spectroscopic data were measured and compared. All investigated ionic liquids have ionic structure, which is confirmed by both the NMR and FT IR spectra. Physico-chemical properties such as specific rotation, thermal stability, glass transition, and surface activity were measured and discussed. Most of the analyzed salts of amino acids are liquids at room temperature. They are thermally stable in the temperature range 150–200 °C. Only 1-ethyl-3-methylimidazolium salts dissolve cellulose.

## Figures and Tables

**Table 1:** ^1^H-NMR and ^13^C-NMR chemical shifts (relative to TMS) of α-positions (H-1′, C-1′) and C=O of compounds studied in DMSO-d_6_ (ppm).

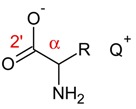			
**Ionic Liquid**	**^1^H-NMR (δ/ppm) (α)**	**^13^C-NMR (δ/ppm) (α)**	**C=O (2′)**
[DDTMA][Gly]	3.09	44.7	181.7
[HDTMA][Gly]	3.25	43.9	179.1
[TBA][l-Val]^a)^	2.59	62.0	176.8
[tBMA][l-Val]	2.56	78.7	176.3
[EMIM][l-Val]	2.63	62.0	177.2
[DDA][l-Val]^b)^	2.55	62.6	176.3
[BA][l-Val]^c)^	3.38	63.8	184.2
[Chol][l-Val]	2.66	61.5	177.4
[HDTMA][l-Val]	3.00	61.6	180.4
[ODTMA][l-Val]	3.00	61.7	180.6
[TBA][l-Leu]^a)^	2.77	56.1	177.8
[tBMA][l-Leu]	2.66	71.1	177.7
[EMIM][l-Leu]	2.75	56.3	178.8
[DDA][l-Leu]^b)^	2.78	62.6	177.7
[BA][l-Leu]^c)^	3.22	64.8	185.7
[Chol][l-Leu]	2.77	54.5	178.7
[DDTMA][l-Leu]	3.25	54.4	181.4
[HDTMA][l-Leu]	3.25	54.2	180.7
[TBA][l-Ile]^a)^	2.63	61.8	176.5
[tBMA][l-Ile]	2.64	78.1	176.4
[EMIM][l-Ile]	2.71	61.7	177.6
[DDA][l-Ile]^b)^	2.62	61.3	176.4
[BA][l-Ile]^c)^	3.14	64.1	185.7
[Chol][l-Ile]	2.72	61.0	177.2
[DDTMA][l-Ile]	3.05	60.9	180.6
[HDTMA][l-Ile]	3.09	61.2	181.8
[TBA][l-Thr]^a)^	2.86	59.1	176.3
[tBMA][l-Thr]	2.92	68.0	175.8
[EMIM][l-Thr]	2.86	59.2	176.6
[DDA][l-Thr]^b)^	2.80	62.6	175.8
[BA][l-Thr]^c)^	3.16	64.2	183.3
[Chol][l-Thr]	2.85	58.4	176.5
[TBA][l-His]^a)^	3.02	56.8	178.9
[tBMA][l-His]	3.93	47.0	176.6
[EMIM][l-His]	3.05	56.9	177.1
[DDA][l-His]^b)^	3.43	62.7	176.6
[BA][l-His]^c)^	3.57	58.1	183.3
[HDTMA][l-His]	3.41	52.8	180.4
[ODTMA][l-His]	3.43	52.8	180.1
[TBA][l-Met]^a)^	2.79	56.0	176.6
[EMIM][l-Met]	2.86	55.5	176.7
[BA][l-Met]^c)^	2.69	58.4	185.8
[Chol][l-Met]	2.88	55.30	177.2
[DDTMA][l-Met]	3.36	55.4	182.2
[HDTMA][l-Met]	3.36	55.4	181.8
[ODTMA][l-Met]	3.20	55.2	181.1
[TBA][l-Trp]^a)^	3.40	58.0	176.9
[EMIM][l-Trp]	3.44	56.0	172.2
[BA][l-Trp]^c)^	3.40	56.5	181.2
[Chol][l-Trp]	2.65	57.0	177.4
[HDTMA][l-Arg]	3.27	55.5	183.2
[ODTMA][l-Arg]	3.15	55.5	183.1
[TBA][l-Tyr]	2.19	58.8	176.8
[BA][l-Tyr]	3.45	58.8	176.8

na – not analyzed/not available; a) Data from [35]; b) Data from [34]; c) Data from [36].

**Table 2:** Selected infrared (IR) bands of amino acid ionic liquids (cm^−1^).

Ionic Liquid	*v*(XH)	*v*(CH_3_-)_as._	*v*(CH_3_-)	*v*(COO^−^)_as._	*v*(COO^−^)_sym._
[DDTMA][Gly]	3600–3000 br.	2918	2850	1578	1396
[HDTMA][Gly]	3600–3000 br.	2918	2850	1585	1407
[TBA][l-Val]^a)^	3600–3100 br.	2959	2874	1570	1397
[DDA][l-Val]^b)^	3500–3100 br.	2956	2854	1573	1397
[BA][l-Val]^c)^	3600–3000 br.	2957	2854	1583	1396
[Chol][l-Val]^d)^	3362	2961	2874	1571	1399
[HDTMA][l-Val]	3600–3000 br.	2920	2851	1584	1396
[ODTMA][l-Val]	3600–3000 br.	2918	2849	1569	1408
[TBA][l-Leu]^a)^	3500–3100br.	2961	2875	1573	1383
[DDA][l-Leu]^b)^	3500–3100 br.	2925	2855	1576	1398
[BA][l-Leu]^c)^	3600–3000 br.	2954	2854	1578	1396
[Chol][l-Leu]^d)^	3377	2956	2871	1578	1401
[DDTMA][l-Leu]	3600–3000 br.	2918	2850	1580	1395
[HDTMA][l-Leu]	3600–3000 br.	2918	2850	1580	1395
[TBA][l-Ile]^a)^	3500–3100br.	2960	2875	1574	1384
[DDA][l-Ile]^b)^	3500–3100 br.	2957	2855	1577	1396
[BA][l-Ile]^c)^	3600–3000 br.	2957	2854	1572	1396
[Chol][l-Ile]^d)^	3378	2963	2876	1575	1402
[DDTMA][l-Ile]	3600–3000 br.	2918	2851	1579	1395
[HDTMA][l-Ile]	3600–3000 br.	2920	2851	1576	1395
[TBA][l-Thr]^a)^	3600–3100br.	2961	2874	1577	1396
[DDA][l-Thr]^b)^	3500–3100 br.	2924	2854	1578	1378
[BA][l-Thr]^c)^	3600–3100 br.	2956	2854	1575	1353
[Chol][l-Thr]^d)^	3366	2973	2933	1577	1402
[TBA][l-His]^a)^	3600–3100 br.	2959	2875	1574	1398
[DDA][l-His]^b)^	3500–3100 br.	2905	2854	1587	1398
[BA][l-His]^c)^	3600–3000 br.	2956	2854	1574	1342
[HDTMA][l-His]	3650–3000 br.	2917	2848	1570	1417
[ODTMA][l-His]	3650–3000 br.	2918	2850	1578	1342
[TBA][l-Met]^a)^	3600–3100 br.	2958	2874	1580	1380
[BA][l-Met]^c)^	3600–3100 br.	2954	2853	1574	1406
[Chol][l-Met]^d)^	3366	2970	2840	1579	1406
[DDTMA][l-Met]	3600–3000 br.	2918	2851	1581	1395
[HDTMA][l-Met]	3700–3000 br.	2920	2851	1581	1391
[ODTMA][l-Met]	3700–3000 br.	2919	2850	1582	1407
[TBA][l-Trp]^a)^	3600–3100 br.	2962	2875	1587	1354
[BA][l-Trp]^c)^	3600–3100 br.	2955	2854	1575	1355
[Chol][l-Trp]^d)^	3252	2921	2874	1573	1404
[HDTMA][l-Arg]	3600–3000 br.	2917	2850	1570	1407
[ODTMA][l-Arg]	3600–3000 br.	2918	2849	1569	1408
[TBA][l-Tyr]	3600–3000 br.	2960	2850	1573	1383
[BA][l-Tyr]	3600–3000 br.	2925	2854	1578	1378

na – not analyzed/not available; a) Data from [35]; b) Data from [34]; c) Data from [36]; d) Data from Supplementary Materials for [26].

**Table 3:** The properties of less-studied AAILs.

No.	Ionic Liquids	State at RT (T_m_ (°C))	Colour	M (g mol^−1^)	T_g_/°C	T_d_^5%^/°C	T_d_^50%^/°C	η/Pa·s 65 °C	[α]_λ_^T^	[M]_λ_^T^	Solubility of Cellulose
1	[DDTMA][Gly]	W	white	303.50	na	160.2 ± 1.1	198.6 ± 1.3	na	-	-	N
2	[HDTMA][Gly]	W	white	358.61	na	177.4 ± 1.9	215.3 ± 1.8	na	-	-	N
3	[TBA][l-Val]	L	colorless	358.61	−53.4 ± 2.1	150.0 ± 1.7	178.9 ± 1.9	0.148±0.031	+4.10	+14.7	N
4	[tBMA][l-Val]	L	colorless	314.20	−55.8 ± 1.9	156.0 ± 2.0	189.0 ± 2.1	1.019±0.056	+4.07	+12.8	N
5	[EMIM][l-Val]	L	colorless	227.32	−73.7 ± 1.2	194.6 ± 2.2	242.2 ± 2.0	1.420±0.073	+6.16	+14.0	Y
6	[DDA][l-Val]	L	colorless	442.78	−23.4 ± 1.6	143.0 ± 1.8	199.5 ± 2.1	0.417±0.009	+2.60	+11.5	N
7	[BA][l-Val]	L	colorless	440.70	−59.6 ± 1.7	149.5 ± 2.1	175.6 ± 1.8	na	+2.69	+11.9	N
8	[Chol][l-Val]	L	colorless	220.32	−74.0^c)^	168.9 ± 1.2	213.3 ± 1.4	372^c)^	+6.81	+15.0	Y
9	[HDTMA][l-Val]	W	white	400.69	na	183.4 ± 1.7	211.8 ± 1.6	na	+3.49	+14.0	N
10	[ODTMA][l-Val]	W	white	429.75	na	177.1 ± 1.6	217.0 ± 1.8	na	+2.48	+10.7	N
11	[TBA][l-Leu]	L	colorless	372.64	−51.8 ± 2.4	153.0 ± 2.1	183.3 ± 1.9	0.149 ± 0.025	+2.44	+9.1	N
12	[tBMA][l-Leu]	L	colorless	328.23	−55.9 ± 2.1	158.8 ± 1.5	189.0 ± 1.7	0.207 ± 0.011	+1.47	+4.8	N
13	[EMIM][l-Leu]	L	colorless	241.35	−60.3 ± 2.5	199.3 ± 1.8	243.8 ± 2.1	4.659 ± 0.073	+2.08	+5.0	Y
14	[DDA][l-Leu]	L	colorless	456.81	−24.7 ± 1.9	134.2 ± 1.1	212.9 ± 0.8	0.596 ± 0.027	+1.78	+8.1	N
15	[BA][l-Leu]	L	colorless	454.73	−55.5 ± 1.6	149.1 ± 0.9	176.0 ± 1.2	na	+1.47	+6.7	N
16	[Chol][l-Leu]	L	colorless	234.35	−47.0^c)^	183.8 ± 0.7	238.1 ± 1.5	476^c)^	+2.80	+6.6	Y
17	[DDTMA][l-Leu]	W	white	359.61	na	182.3 ± 2.0	196.4 ± 2.4	na	+0.96	+3.5	N
18	[HDTMA][l-Leu]	W	white	414.71	na	175.7 ± 1.8	211.0 ± 1.4	na	+2.96	+12.3	N
19	[TBA][l-Ile]	L	colorless	372.64	−53.2 ± 1.3	146.5 ± 1.6	177.8 ± 1.8	0.176 ± 0.032	+3.91	+14.6	N
20	[tBMA][l-Ile]	L	colorless	328.23	−55.7 ± 2.1	157.4 ± 1.8	189.0 ± 1.4	3.880 ± 0.041	+3.75	+12.3	N
21	[EMIM][l-Ile]	L	colorless	241.35	−61.8 ± 2.4	198.2 ± 2.2	246.7 ± 2.3	3.499 ± 0104	+5.78	+14.0	Y
22	[DDA][l-Ile]	L	colorless	456.81	−23.3 ± 1.8	137.3 ± 2.1	191.5 ± 1.6	0.655 ± 0.028	+2.80	+12.8	N
23	[BA][l-Ile]	L	colorless	454.73	−63.9 ± 1.7	147.3 ± 1.9	174.0 ± 1.5	na	+2.54	+11.6	N
24	[Chol][l-Ile]	L	colorless	234.35	−47.0^c)^	190.8 ± 1.3	239.7 ± 1.0	480^c)^	+6.07	+14.2	Y
25	[DDTMA][l-Ile]	W	white	359.61	na	166.4 ± 1.9	193.8 ± 1.3	na	+1.29	+4.6	N
26	[HDTMA][l-Ile]	W	white	414.71	na	173.6 ± 1.7	207.5 ± 2.5	na	+5.27	+21.9	N
27	[TBA][l-Thr]	L	colorless	360.58	−40.0 ± 2.1	163.0 ± 1.5	193.7 ± 2.1	0.316 ± 0.002	-2.46	-8.9	N
28	[tBMA][l-Thr]	L	colorless	316.17	−47.5 ± 2.3	163.5 ± 1.4	200.9 ± 2.2	0.626 ± 0.038	-1.47	-4.6	N
29	[EMIM][l-Thr]	L	colorless	229.29	−55.1 ± 1.4	169.1 ± 1.5	198.9 ± 1.2	0.129 ± 0.004	-3.7	-8.9	Y
30	[DDA][l-Thr]	L	colorless	444.75	−58.7 ± 1.7	158.8 ± 2.1	204.2 ± 1.7	0.548 ± 0.007	−1.78	−7.9	N
31	[BA][l-Thr]	L	colorless	442.67	−55.2 ± 1.3	153.6 ± 2.2	178.3 ± 1.6	na	−2.57	−11.4	N
32	[Chol][l-Thr]	L	colorless	222.29	−39.0^c)^	194.9 ± 1.6	240.2 ± 1.9	454^c)^	−3.05	−6.8	Y
33	[TBA][l-His]	S (128.5 ± 2.1)	yellow	396.62	−17.1 ± 1.2	181.6 ± 1.4	234.7 ± 1.5	-	−3.90	−15.5	N
34	[tBMA][l-His]	S (58.9 ± 1.2)	yellow	352.21	−42.6 ± 1.5	173.4 ± 2.2	226.4 ± 2.6	-	−3.33	−11.7	N
35	[EMIM][l-His]	W	yellow	265.33	−33.4 ± 2.2	216.4 ± 1.7	315.0 ± 1.2	0.497 ± 0.071	−5.07	−13.5	Y
36	[DDA][l-His]	L	yellow	480.79	−39.0 ± 0.4	170.5 ± 1.4	249.6 ± 1.9	1.604 ± 0.008	−3.62	−17.4	N
37	[BA][l-His]	L	yellow	478.71	−39.8 ± 0.3	140.3 ± 0.5	215.8 ± 1.3	na	−3.89	−18.6	N
38	[HDTMA][l-His]	W	white	438.70	na	171.9 ± 1.0	243.9 ± 1.6	na	−6.00	−26.3	N
39	[ODTMA][l-His]	W	white	467.76	na	212.2 ± 1.3	235.4 ± 2.1	na	−4.92	−23.0	N
40	[TBA][l-Met]	L	pale yellow	390.67	−57.9 ± 1.1	155.3 ± 1.8	185.0 ± 2.1	na	+1.20	+4.7	N
41	[EMIM][l-Met]	L	yellow	259.38	−57.0 ± 1.7	194.0 ± 2.1	238.0 ± 1.6	na	+1.41	+3.7	Y
42	[BA][l-Met]	L	colorless	472.76	−61.0 ± 1.5	148.5 ± 1.9	183.1 ± 1.3	na	+2.01	+9.5	N
43	[Chol][l-Met]	W	pale yellow	252.38	−61.0^c)^	179.9 ± 1.3	213.1 ± 1.6	330^c)^	+2.30	+5.8	Y
44	[DDTMA][l-Met]	W	white	377.64	na	170.3 ± 2.4	195.5 ± 1.9	na	+4.00	+15.1	N
45	[HDTMA][l-Met]	W	white	432.76	na	173.6 ± 1.7	214.6 ± 1.5	na	+1.28	+5.6	N
46	[ODTMA][l-Met]	W	white	461.81	na	176.7 ± 0.4	224.9 ± 1.3	na	+0.65	+3.0	N
47	[TBA][l-Trp]	S (129.6 ± 2.3)	creamy beige	445.68	-	181.7 ± 1.8	234.2 ± 0.9	-	ch−13.1^a)^	-−58.4^a)^	N
48	[EMIM][l-Trp]	L	brown	314.40	−31.0 ± 2.1	191.5 ± 2.5	310.4 ± 2.1	na	−1.65	−5.2	Y
49	[BA][l-Trp]	W	brown	527.78	−28.3 ± 1.9	145.0 ± 2.3	206.3 ± 1.7	na	−2.54	−13.4	N
50	[Chol][l-Trp]	L	dark brown	307.40	−12.0^c)^	187.6 ± 1.2	239.2 ± 1.8	5640^c)^	−2.55	−7.8	Y
51	[HDTMA][l-Arg]	W	white	457.74	na	184.4 ± 1.4	252.5 ± 1.6	na	+6.34	+29.0	N
52	[ODTMA][l-Arg]	W	white	486.80	na	186.9 ± 2.1	235.6 ± 2.0	na	+4.34	+21.1	N

na—not analyzed/not available; L—liquid, S—solid, W—wax; Y—ability to dissolve of cellulose, N—lack of ability to dissolve of cellulose; a) results for ethanol; b) data from [1]; c) data from [25]; viscosity at 20 °C

**Table 4:** Miscibility with other solvents of amino acid ionic liquids [TBA][AA].

Ionic Liquids	Water (9.0)	Acetone (5.4)	Ethanol (5.2)	Chloroform (4.4)	Ethyl Acetate (4.3)	Benzene (3.0)	Diethyl Ether (2.9)	*N–H*exane (0.0)
[DDTMA][Gly]	m	p	p	i	i	i	i	i
[HDTMA][Gly]	m	p	p	i	i	i	i	i
[TBA][l-Val]	m	m	m	m	m	i	i	i
[tBMA][l-Val]	m	m	m	m	m	i	i	i
[EMIM][l-Val]	m	m	m	m	m	i	i	i
[DDA][l-Val]	m	m	m	m	m	i	i	i
[BA][l-Val]	m	m	m	m	m	i	i	i
[Chol][l-Val]	m	m	m	m	m	i	i	i
[HDTMA][l-Val]	m	p	p	i	i	i	i	i
[ODTMA][l-Val]	m	m	m	i	i	i	i	i
[TBA][l-Leu]	m	m	m	m	m	i	i	i
[tBMA][l-Leu]	m	m	m	m	m	i	i	i
[EMIM][l-Leu]	m	m	m	m	m	i	i	i
[DDA][l-Leu]	m	m	m	m	m	i	i	i
[BA][l-Leu]	m	m	m	m	m	i	i	i
[Chol][l-Leu]	m	m	m	m	m	i	i	i
[DDTMA][l-Leu]	m	m	m	m	i	i	i	i
[HDTMA][l-Leu]	m	m	m	m	i	i	i	i
[TBA][l-Ile]	m	m	m	m	m	i	i	i
[tBMA][l-Ile]	m	m	m	m	m	i	i	i
[EMIM][l-Ile]	m	m	m	m	m	i	i	i
[DDA][l-Ile]	m	m	m	m	m	i	i	i
[BA][l-Ile]	m	m	m	m	m	i	i	i
[Chol][l-Ile]	m	m	m	m	m	i	i	i
[DDTMA][l-Ile]	m	m	m	m	i	i	i	i
[HDTMA][l-Ile]	m	m	m	m	i	i	i	i
[TBA][l-Thr]	m	m	m	p	m	i	i	i
[tBMA][l-Thr]	m	m	m	m	m	i	i	i
[EMIM][l-Thr]	m	m	m	m	m	i	i	i
[DDA][l-Thr]	m	m	m	m	m	i	i	i
[BA][l-Thr]	m	m	m	m	m	i	i	i
[Chol][l-Thr]	m	m	m	m	m	i	i	i
[TBA][l-His]	m	m	m	m	p	i	i	i
[tBMA][l-His]	m	m	m	p	p	i	i	i
[EMIM][l-His]	m	m	m	m	m	i	i	i
[DDA][l-His]	m	m	m	m	p	i	i	i
[BA][l-His]	m	m	m	m	m	i	i	i
[HDTMA][l-His]	m	m	p	i	i	i	i	i
[ODTMA][l-His]	m	m	m	i	i	i	i	i
[TBA][l-Met]	m	m	m	m	m	i	i	i
[EMIM][l-Met]	m	m	m	m	m	i	i	i
[BA][l-Met]	m	m	m	m	m	i	i	i
[Chol][l-Met]	m	m	m	m	m	i	i	i
[DDTMA][l-Met]	m	m	m	m	i	i	i	i
[HDTMA][l-Met]	m	m	m	m	i	i	i	i
[ODTMA][l-Met]	m	m	m	i	i	i	i	i
[TBA][l-Trp]	m	m	m	p	p	i	i	i
[EMIM][l-Trp]	m	m	m	m	m	i	i	i
[BA][l-Trp]	m	m	m	m	m	i	i	i
[Chol][l-Trp]	m	m	m	p	p	i	i	i
[TBA][l-Tyr]	m	m	m	m	m	i	i	i
[BA][l-Tyr]	m	m	m	m	m	i	i	i
[HDTMA][l-Arg]	m	p	m	i	i	i	i	i
[ODTMA][l-Arg]	m	m	m	i	i	i	i	i

m—miscible; p—partly miscible; i—immiscible.

**Table 5:** Critical micellar concentration (CMC), surface tension (σ_CMC_), surface excess concentration (Γ_max_), and area per molecule (A_min_) of the prepared ILs in aqueous solution at 25 °C.

IL	CMC (mmol·L^−1^)	ϒ_CMC_ (mN·m^−1^)	Γ_max_·10^6^ (mol·m^−2^)	A_min_·10^−19^ (m^2^)	*p*C_20_ (mmol·L^−1^)
[DDA][l-Val]^a)^	0.64	28.3	3.302	5.186	0.029
[DDA][l-Leu]^a)^	0.61	27.9	3.364	4.936	0.021
[DDA][l-Ile]^a)^	0.60	28.0	3.373	4.932	0.014
[DDA][l-Thr]^a)^	0.87	28.2	2.922	5.683	0.018
[DDA][l-His]^a)^	0.82	29.4	3.071	5.407	0.025
[BA][l-Val]^b)^	0.28	32.3	4.853	3.423	0.056
[BA][l-Leu]^b)^	0.25	32.9	5.645	2.943	0.063
[BA][l-Ile]^b)^	0.26	32.8	5.654	2.938	0.062
[BA][l-Thr]^b)^	0.31	32.8	7.151	2.322	0.048
[BA][l-His]^b)^	0.28	34.1	5.604	2.964	0.061
[BA][l-Met]^b)^	0.25	34.8	11.751	1.414	0.055
[BA][l-Trp]^b)^	0.34	33.7	7.463	2.226	0.068
[DDTMA][Gly]	12.26	40.3	5.471	3.035	6.386
[DDTMA][l-Leu]	11.81	40.3	5.273	3.103	3.621
[DDTMA][l-Ile]	12.17	40.2	5.283	3.143	3.599
[DDTMA][l-Met]	12.09	40.4	7.561	2.196	2.645
[HDTMA][Gly]	0.78	41.3	5.273	3.149	0.356
[HDTMA][l-Val]	0.78	41.7	8.157	2.036	0.316
[HDTMA][l-Leu]	0.76	40.8	5.663	2.892	0.271
[HDTMA][l-Ile]	0.74	40.7	5.753	2.887	0.264
[HDTMA][l-Met]	0.86	41.8	4.179	3.974	0.187
[HDTMA][l-His]	0.96	41.7	5.870	2.829	0.372
[HDTMA][l-Arg]	1.09	41.7	8.040	2.065	0.165
[ODTMA][l-Val]	0.53	40.2	3.281	5.062	0.015
[ODTMA][l-Met]	0.52	40.3	2.503	6.635	0.007
[ODTMA][l-His]	0.50	42.0	2.903	5.720	0.011
[ODTMA][l-Arg]	0.61	40.1	3.492	4.755	0.008

a)Data from [30]; b)Data from [34].

**Table 6:** The solubility of cellulose in [EMIM][AA] and [Chol][AA] ILs.

Ionic Liquid	Cellulose (mg g^−1^)
[EMIM][l-Val]	21.6 (60 °C)
[EMIM][l-Leu]	34.4 (60 °C)
[EMIM][l-Ile]	13.7 (60 °C)
[EMIM][l-Thr]	43.2 (60 °C)
[EMIM][Cl]	31.4 (85 °C)
[Chol][l-Val]^a)^	<5 (90 °C)
[Chol][l-Leu]^a)^	<5 (90 °C)
[Chol][l-Ile]^a)^	<5 (90 °C)
[Chol][l-Thr]^a)^	<5 (90 °C)
[Chol][l-His]^a)^	<5 (90 °C)
[Chol][l-Met]^a)^	<5 (90 °C)
[Chol][l-Trp]^a)^	<5 (90 °C)
[Chol][l-Tyr]^a)^	<5 (90 °C)

a) Data from [25].

## Supplementary Materials

The following are available online at https://www.mdpi.com/1420-3049/24/18/3252/s1.
