# Supplementary material for: The Relationship between the Structure and Properties of Amino Acid Ionic Liquids
Source: Molecules. 2019 Sep 6;24(18):3252. doi: 10.3390/molecules24183252 (PMC6767286; doi:10.3390/molecules24183252)
Supplement: Supplementary file 1 [file molecules-24-03252-s001.pdf]

The relationship between the structure and properties of Amino Acid Ionic Liquids

Paula Ossowicz[a*], Joanna Klebeko[a], Barbara Roman[a], Ewa Janus[a], Zbigniew Rozwadowski[b]

a) Department of Organic Chemical Technology, Faculty of Chemical Technology and Engineering, West Pomeranian University of Technology, Szczecin, ul. Pułaskiego 10, Szczecin 70-322, Poland
b) Department of Inorganic and Analytical Chemistry, Faculty of Chemical Technology and Engineering, West Pomeranian University of Technology, Al. Piastów 42, Szczecin 71-065, Poland

**Corresponding author: Paula Ossowicz, e-mail: possowicz@zut.edu.pl; phone: +48914494801*

SUPPLEMENTARY DATA

General Synthetic Methods The ^{1}H and ^{13}C NMR spectra for obtained amino acid ionic liquids were recorded on a BRUKER DPX-400 spectrometer operating at 400 MHz (^{1}H) and 100 MHz (^{13}C) as solutions in D_2O or DMSO. Chemical shifts are given in δ (ppm) and coupling constants *J* are given in Hz. Infrared spectra were recorded on Nicolet FT-IR 380 (Thermo Electron Corporation) in transmission mode on KBr pellets and are reported in wave numbers (cm^{-1}).

Tributylmethylammonium L-valinate [tBMA][L-Val]:
^{1}H NMR (400 MHz, DMSO-d_6) δ, ppm: 0.64 (d, 3H, J = 6.8 Hz, CH_3-Val); 0.78 (d, 3H, J = 6.9 Hz, CH_3-Val); 0.92 (t, 9H, J = 7.4 Hz, CH_3-cation); 1.24-1.34 (m, 6H, -CH_2- cation); 1.55-1.63 (m, 6H, -CH_2- cation); 1.83-1.90 (m, 1H, CH-Val); 2.56 (ov, 1H, -CH-Val); 2.94 (s, 3H, CH_3-cation); 3.19 (t, 6H, J = 8.4 Hz, -CH_2-cation); ^{13}C NMR (100 MHz, DMSO-d_6) δ, ppm: 13.92 (CH_3- cation); 18.58 (CH_3-Val); 19.66 (CH_3-Val); 20.64 (-CH_2-cation); 23.55 (-CH_2-cation); 31.43 (CH-Val); 45.92 (CH_3-cation); 57.97 (-CH_2-cation); 78.70 (CH-Val); 176.26 (-COO^- Val).

Tributylmethylammonium L-leucinate [tBMA][L-Leu]:
^{1}H NMR (400 MHz, DMSO-d_6) δ, ppm: 0.77 (d, 3H, J = 6.6 Hz, CH_3-Leu); 0.82 (d, 3H, J = 6.6 Hz, CH_3-Leu); 0.92 (t, 9H, J = 7.4 Hz, CH_3-cation); 1.24-1.34 (m, 6H, -CH_2-cation); 1.35-1.40 (m,

1H, -CH-Leu); 1.53-1.57 (m, 6H, -CH_2-cation); 1.63-1.66 (m, 1H, -CH_2-Leu); 2.66 (ov, 1H, -CH-Leu); 2.98 (s, 3H, CH_3-cation); 3.23 (t, 6H, J = 8.4 Hz, -CH_2-cation); ^{13}C NMR (100 MHz, DMSO-d_6) δ, ppm: 13.95 (CH_3-cation); 19.67 (-CH_2- cation); 22.82 (CH_3-Leu); 23.12 (CH_3-Leu); 23.55 (-CH_2-cation); 25.20 (CH-Leu); 43.62 (CH_2-Leu); 45.96 (CH_3-cation); 57.99 (-CH_2-cation); 71.05 (-CH- Leu); 177.72 (-COO^- Leu).

Tributylmethylammonium L-isoleucinate [tBMA][L-Ile]:

^{1}H NMR (400 MHz, DMSO-d_6) δ, ppm: 0.74 (d, 3H, J = 7.6 Hz, CH_3-Ile); 0.93 (t, 9H, J = 7.5 Hz, CH_3-cation); 0.99 (t, 3H, J = 8.2 Hz, CH_3-Ile); 1.22-1.36 (m, 8H, -CH_2-cation, -CH_2-Ile); 1.53-1.58 (m, 7H, -CH_2-cation, -CH-Ile); 2.64 (d, 1H, CH-Ile); 2.94 (s, 3H, CH_3-cation); 3.23 (t, 6H, J = 8.0 Hz, -CH_2-cation); ^{13}C NMR (100 MHz, DMSO-d_6) δ, w ppm: 12.04 (CH_3-CH_2-Ile); 12.10 (CH_3-CH_2- cation); 15.64 (CH_3-CH_2-Ile); 19.67 (CH_3-CH_2 cation); 23.55 (-CH_2- cation); 31.43 (CH_3-CH-Ile); 38.06 (CH-CH_3-Ile); 45.93 (CH_3-N^+ cation); 57.97 (-CH_2-N^+ cation); 78.17 (CH-COO^- Ile); 176.40 (-COO^- Ile).

Tributylmethylammonium L-threoninate [tBMA][L-Thr]:

^{1}H NMR (400 MHz, DMSO-d_6) δ, ppm: 0.98 (t, 9H, J = 4.0 Hz, CH_3-cation); 1.31 (d, 3H, J = 7.4 Hz, CH_3-Thr); 1.31-1.40 (m, 6H, -CH_2-cation); 1.62-1.70 (m, 6H, -CH_2-cation); 3.05 (s, 3H, CH_3-cation); 3.36 (t, 6H, J = 8.4 Hz, CH_2-cation); 2.92 (d, 1H, J = 4.9 Hz, CH-Thr); 3.54-3.60 (m, 1H, CH-Thr); ^{13}C NMR (100 MHz, DMSO-d_6) δ, ppm: 13.94 (CH_3-cation); 19.67 (-CH_2-cation); 20.15 (CH_3-Thr); 23.53 (-CH_2-cation); 45.97 (CH_3-cation); 57.98 (-CH_2-cation); 67.97 (-CH-Thr); 77.07 (CH-Thr); 175.76 (-COO^- Thr).

Tributylmethylammonium L-histidinate [tBMA][L-His]:

^{1}H NMR (400 MHz, DMSO-d_6) δ, ppm: 0.98 (t, 9H, J = 7.4 Hz, CH_3-cation); 1.31-1.40 (m, 6H, -CH_2-cation); 1.62-1.70 (m, 6H, -CH_2-cation); 2.50 (d, 1H, J = 1.6 Hz, CH(H)-His); 2.90 (d, 1H, J = 10.7 Hz, CH(H)-His); 3.04 (s, 3H, CH_3-cation); 3.29 (t, 6H, J = 8.4 Hz, CH_2-cation); 3.93 (t, 1H, CH-His); 6.68 (s, 1H, CH(Ar)-His); 7.46 (s, 1H, CH(Ar)-His); ^{13}C NMR (100 MHz, DMSO-d_6) δ, ppm: 13.91 (CH_3-cation); 19.65 (-CH_2-cation); 23.56 (-CH_2-cation); 29.00 (CH_2-His); 47.01 (CH-His); 57.99 (-CH_2-cation); 116.99 (CH(Ar)-His); 134.18 (CH(Ar)-His); 136.33 (CH(Ar)-His); 176.59 (-COO^- His).

Dodecyltrimethylammonium glycinate ([DDTMA][Gly]):

^{1}H NMR (400 MHz, D_2O) δ, ppm: 0.85-0.90 (t, 3H, CH_3-DDTMA, d, 6H, CH_3-DDTMA); 1.16-1.34 (m, 18H, -CH_2-DDTMA); 1.63-1.73 (m, 2H, -CH_2-DDTMA); 3,03 (s, 9H, CH_3-DDTMA); 3.09 (s, 2H, -CH_2-Gly); 3.21 (t, 2H, J = 8.7 Hz, -CH_2-DDTMA); ^{13}C NMR (100 MHz, D_2O) δ, ppm: 13.89 (CH_3-DDTMA); 22.60 (-CH_2-DDTMA); 25.95 (-CH_2-DDTMA); 28.91 (-CH_2-DDTMA); 29.31 (-CH_2-DDTMA); 29.46 (-CH_2-DDTMA); 29.59 (-CH_2-DDTMA); 31.87 (-CH_2-DDTMA); 44.74 (-CH_2-Gly); 52.87 (-CH_3 DDTMA); 66.70 (-CH_2-DDTMA); 181.69 (-COO^- Gly); FT-IR ν_{max} (KBr, thin film): 3600-3000 (br), 2918, 2850, 1578, 1487, 1472, 1396, 1298, 963, 911, 720 cm^{-1};

Dodecyltrimethylammonium L-leucinate ([DDTMA][L-Leu]):

^{1}H NMR (400 MHz, D_2O) δ, ppm: 0.74 (t, 3H, J = 6.9 Hz, CH_3-DDTMA); 0.78 (d, 3H, J = 6.6 Hz, CH_3-Leu); 0.80 (d, 3H, J = 6.6 Hz, CH_3-Leu); 1.10-1.25 (m, 18H, -CH_2-DDTMA); 1.29-1.34 (m, 1H, -CH-Leu); 1.40-1.44 (m, 1H, -CH(H)-Leu); 1.52-1.56 (m, 1H, -CH(H)-Leu); 1.60-1.66 (m, 2H, -CH_2-DDTMA); 2.98 (s, 9H, CH_3-DDTMA); 3.16 (t, 2H, J = 8.7 Hz, -CH_2-DDTMA); 3.25 (t, 1H, J = 8.7 Hz, -CH-Leu); ^{13}C NMR (100 MHz, D_2O) δ, ppm: 13.88 (CH_3-HDTMA); 21.43 (-CH_3 Leu); 21.87 (-CH_3 Leu); 22.58 (-CH_2-HDTMA); 22.67 (-CH-Leu); 24.54 (-CH_2-HDTMA); 26.01 (-CH_2-HDTMA); 28.94 (-CH_2-HDTMA); 29.27 (-CH_2-HDTMA); 28.27 (-CH_2-HDTMA); 29.32 (-CH_2-HDTMA); 29.45 (-CH_2-HDTMA); 29.56 (-CH_2-HDTMA); 29.58 (-CH_2-HDTMA); 31.84 (-CH_2-HDTMA); 43.13 (-CH-Leu); 52.96 (-CH_3 HDTMA); 54.38 (-CH-Leu); 66.75 (-CH_2-HDTMA); 181.37 (-COO^- Leu); FT-IR ν_{max} (KBr, thin film): 3600-3000 (br), 2918, 2850, 1580, 1514, 1468, 1395, 964, 911, 720 cm^{-1};

Dodecyltrimethylammonium L-isoleucinate ([DDTMA][L-Ile]):

^{1}H NMR (400 MHz, D_2O) δ, ppm: 0.84-0.90 (d, 6H, CH_3-Ile, t, 3H, CH_3-DDTMA); 1.10-1.15 (m, 1H, -CH(H)-Ile); 1.24-1.38 (m, 18H, -CH_2-DDTMA); 1.39-1.45 (m, 1H, -CH(H)-Ile); 1.60-1.68 (m, 1H, -CH-Ile); 1.71-1.79 (m, 2H, -CH_2-DDTMA); 3.05 (d, 1H, J = 5.4 Hz, -CH-Ile); 3.10 (s, 9H, CH_3-DDTMA); 3.28 (t, 2H, J = 8.6 Hz, -CH_2-DDTMA); ^{13}C NMR (100 MHz, D_2O) δ, ppm: 11.29 (CH_3-Ile); 13.89 (CH_3-DDTMA); 15.56 (-CH_2-Ile); 22.64 (-CH_2-DDTMA); 22.71 (-CH_2-DDTMA); 24.29 (CH_3-Ile); 26.10 (-CH_2-DDTMA); 29.11 (-CH_2-DDTMA); 29.42 (-CH_2-

DDTMA); 29.53 (-CH_2-DDTMA); 29.65 (-CH_2-DDTMA); 29.73 (-CH_2-DDTMA); 29.78 (-CH_2-DDTMA); 31.94 (-CH_2-DDTMA); 38.33 (-CH-Ile); 52.89 (-CH_3 DDTMA); 60.89 (-CH-Ile); 66.59 (-CH_2-DDTMA); 180.58 (-COO^- Ile); FT-IR ν_{max} (KBr, thin film): 3600-3000 (br), 2918, 2851, 1579, 1513, 1487, 1464, 1395, 964, 911, 719 cm^{-1};

Dodecyltrimethylammonium L-methioninate ([DDTMA][L-Met]):

^1H NMR (400 MHz, D_2O) δ, ppm: 0.94 (t, 3H, J = 6.4 Hz, CH_3-DDTMA); 1.29-1.46 (m, 18H, -CH_2-DDTMA); 1.79-1.87 (m, 2H, -CH_2-DDTMA, m, 1H, CH(H)-Met); 1.94-2.03 (m, 1H, CH(H)-Met); 2.16 (s, 3H, CH_3-Met); 2.61 (t, 2H, J = 7,8 Hz, -CH_2-Met); 3.18 (s, 9H, CH_3-DDTMA); 3.34-3.38 (t, 2H, -CH_2-DDTMA, t, 1H, -CH-Met); ^{13}C NMR (100 MHz, D_2O) δ, ppm: 13.93 (CH_3-DDTMA); 14.33 (-CH_3 Met); 22.64 (-CH_2-DDTMA); 22.70 (-CH_2-DDTMA); 26.07 (-CH_2-DDTMA); 29.06 (-CH_2-DDTMA); 29.38 (-CH_2-DDTMA); 29.45 (-CH_2-DDTMA); 29.58 (-CH_2-DDTMA); 29.67 (-CH_2-DDTMA); 30.02 (-CH_2-DDTMA); 30.06 (-CH_2-Met); 31.92 (-CH_2-DDTMA); 34.45 (-CH_2-Met); 52.89 (-CH_3 DDTMA); 55.42 (-CH-Met); 66.63 (-CH_2-DDTMA); 182.22 (-COO^- Met); FT-IR ν_{max} (KBr, thin film): 3600-3000 (br), 2918, 2851, 1581, 1509, 1487, 1464, 1395, 964, 720 cm^{-1};

Hexadecylltrimethylammonium glycinate ([HDTMA][Gly]):

^1H NMR (400 MHz, D_2O) δ, ppm: 0.92 (t, 3H, J = 6.0 Hz, CH_3-HDTMA); 1.28-1.42 (m, 26H, -CH_2-HDTMA); 1.73-1.83 (m, 2H, -CH_2-HDTMA); 3.14 (s, 9H, CH_3-HDTMA); 3.25 (s, 2H, -CH_2-Gly); 3.32 (t, 2H, -CH_2-HDTMA); ^{13}C NMR (100 MHz, D_2O) δ, ppm: 13.88 (CH_3-HDTMA); 22.63 (-CH_2-HDTMA); 26.02 (-CH_2-HDTMA); 29.06 (-CH_2-HDTMA); 29.51 (-CH_2-HDTMA); 29.68 (-CH_2-HDTMA); 29.78 (-CH_2-HDTMA); 29.87 (-CH_2-HDTMA); 29.89 (-CH_2-HDTMA); 31.96 (-CH_2-HDTMA); 43.90 (-CH_2-Gly); 52.81 (-CH_3 HDTMA); 66.53 (-CH_2-HDTMA); 179.07 (-COO^- Gly); FT-IR ν_{max} (KBr, thin film): 2918, 2850, 1585, 1487, 1473, 1463, 1407, 1334, 1303, 961, 912, 829 cm^{-1};

Hexadecylltrimethylammonium L-valinate ([HDTMA][L-Val]):

^1H NMR (400 MHz, D_2O) δ, ppm: 0.76 (d, 3H, CH_3-Val, t, 3H, CH_3-HDTMA); 0.81 (d, 3H, J = 7.1 Hz, CH_3-Val); 1.09-1.30 (m, 26H, -CH_2-HDTMA); 1.57-1.67 (m, 2H, -CH_2-HDTMA); 1.83-1.88 (m, 1H, -CH-Val); 2.99 (d, 1H, -CH-Val; s, 9H, CH_3-HDTMA); 3.17 (t, 2H, J = 8.4 Hz, -

CH_2-HDTMA); ^{13}C NMR (100 MHz, D_2O) δ, ppm: 13.86 (CH_3-HDTMA); 16.81 (-CH_3 Val); 18.99 (-CH_3 Val); 22.63 (-CH_2-HDTMA); 22.67 (-CH_2-HDTMA); 26.09 (-CH_2-HDTMA); 29.16 (-CH_2-HDTMA); 29.48 (-CH_2-HDTMA); 29.81 (-CH_2-HDTMA); 29.93 (-CH_2-HDTMA); 29.96 (-CH_2-HDTMA); 31.25 (-CH-Val); 31.97 (-CH_2-HDTMA); 52.82 (-CH_3 HDTMA); 61.55 (-CH-Val); 66.45 (-CH_2-HDTMA); 180.35 (-COO^-Val); FT-IR ν_{max} (KBr, thin film): 3600-3100 (br), 2920, 2851, 1584, 1507, 1464, 1396, 1329, 964, 911, 718 cm^{-1};

Hexadecylltrimethylammonium L-leucinate ([HDTMA][L-Leu]):

^1H NMR (400 MHz, D_2O) δ, ppm: 0.75 (t, 3H, J = 7.1 Hz, CH_3-HDTMA); 0.79 (d, 3H, J = 6.6 Hz, CH_3-Leu); 0.81 (d, 3H, J = 6.6 Hz, CH_3-Leu); 1.12-1.27 (m, 26H, -CH_2-HDTMA); 1.30-1.34 (m, 1H, -CH-Leu); 1.42-1.45 (m, 1H, -CH(H)-Leu); 1.52-1.57 (m, 1H, -CH(H)-Leu); 1.59-1.65 (m, 2H, -CH_2-HDTMA); 2.99 (s, 9H, CH_3-HDTMA); 3.17 (t, 2H, J = 8,6 Hz, -CH_2-HDTMA); 3.25 (t, 1H, J = 8,8 Hz, -CH-Leu); ^{13}C NMR (100 MHz. D_2O) δ. ppm: 13.87 (CH_3-HDTMA); 21.37 (-CH_3 Leu); 22.61 (-CH_3 Leu); 22.65 (-CH_2-HDTMA); 22.74 (-CH-Leu); 24.48 (-CH_2-HDTMA); 26.18 (-CH_2-HDTMA); 29.25 (-CH_2-HDTMA); 29.50 (-CH_2-HDTMA); 29.70 (-CH_2-HDTMA); 29.84 (-CH_2-HDTMA); 29.93 (-CH_2-HDTMA); 29.97 (-CH_2-HDTMA); 30.00 (-CH_2-HDTMA); 31.99 (-CH_2-HDTMA); 43.14 (-CH-Leu); 52.87 (-CH_3 HDTMA); 54.24 (-CH-Leu); 66.48 (-CH_2-HDTMA); 180.69 (-COO^- Leu); FT-IR ν_{max} (KBr, thin film): 3600-3000 (br), 2918, 2850, 1580, 1515, 1463, 1395, 964, 912, 719 cm^{-1};

Hexadecylltrimethylammonium L-isoleucinate ([HDTMA][Gly]):

^1H NMR (400 MHz, D_2O) δ, ppm: 0.90-0.92 (d, 3H, J = 8.0 Hz, CH_3-Ile, t, 6H, J = 4.0 Hz, CH_3-Ile, CH_3-HDTMA); 1.12-1.19 (m, 1H, -CH(H)-Ile); 1.33-1.39 (m, 26H, -CH_2-HDTMA); 1.47-1.50 (m, 1H, -CH(H)-Ile); 1.63-1.70 (m, 1H, -CH-Ile); 1.74-1.85 (m, 2H, -CH_2-HDTMA); 3.09 (t, 1H, J = 8.0 Hz, -CH-Ile); 3,16 (s, 9H, CH_3-HDTMA); 3.34 (t, 2H, J = 8.0 Hz, -CH_2-HDTMA); ^{13}C NMR (100 MHz. D_2O) δ. ppm: 11.32 (CH_3-Ile); 13.86 (CH_3-HDTMA); 15.70 (-CH_2-Ile); 22.64 (-CH_2-HDTMA); 22.70 (-CH_2-HDTMA); 24.26 (CH_3-Ile); 26.13 (-CH_2-HDTMA); 29.20 (-CH_2-HDTMA); 29.48 (-CH_2-HDTMA); 29.67 (-CH_2-HDTMA); 29.81 (-CH_2-HDTMA); 29.91 (-CH_2-HDTMA); 29.97 (-CH_2-HDTMA); 31.97 (-CH_2-HDTMA); 38.88 (-CH-Ile); 52.82 (-CH_3 HDTMA); 61.15 (-CH-Ile); 66.46 (-CH_2-HDTMA); 181.82 (-COO^- Ile); FT-IR ν_{max} (KBr, thin film): 3600-3000 (br), 2920, 2851, 1576, 1513, 1464, 1395, 964, 911, 720 cm^{-1};

Hexadecylltrimethylammonium L-methioninate ([HDTMA][L-Met]):

^{1}H NMR (400 MHz, D_2O) δ, ppm: 0.87 (t, 3H, J = 6.0 Hz, CH_3-HDTMA); 1.24-1.42 (m, 26H, -CH_2-HDTMA); 1.71-1.78 (m, 2H, -CH_2-HDTMA); 1.79-1.84 (m, 1H, CH(H)-Met); 1.92-1.97 (m, 1H, CH(H)-Met); 2.08 (s, 3H, CH_3-Met); 2.55 (t, 2H, J = 8.0 Hz, -CH_2-Met); 3.11 (s, 9H, CH_3-HDTMA); 3.29 (t, 2H, J = 8.0 Hz, -CH_2-HDTMA); 3.36 (t, 1H, J=4.0 Hz, -CH-Met); ^{13}C NMR (100 MHz, D_2O) δ, ppm: 13.86 (CH_3-HDTMA); 14.36 (-CH_3 Met); 22.65 (-CH_2-HDTMA); 22.73 (-CH_2-HDTMA); 26.17 (-CH_2-HDTMA); 29.24 (-CH_2-HDTMA); 29.49 (-CH_2-HDTMA); 29.69 (-CH_2-HDTMA); 29.83 (-CH_2-HDTMA); 29.93 (-CH_2-HDTMA); 29.96 (-CH_2-HDTMA); 29.99 (-CH_2-Met); 31.98 (-CH_2-HDTMA); 34.49 (-CH_2-Met); 52.83 (-CH_3 HDTMA); 55.37 (-CH-Met); 66.48 (-CH_2-HDTMA); 181.77 (-COO^- Met); FT-IR ν_{max} (KBr, thin film): 3700-3000 (br), 2920, 2851, 1581, 1510, 1465, 1391, 965, 911, 720 cm^{-1};

Hexadecylltrimethylammonium L-histidinate ([HDTMA][L-His]):

^{1}H NMR (400 MHz, D_2O) δ, ppm: 0.75 (t, 3H, J = 6.4 Hz, CH_3-HDTMA); 1.06-1.26 (m, 26H, -CH_2-HDTMA); 1.52-1.67 (m, 2H, -CH_2-HDTMA); 2.70 (d, 1H, J = 7.8 Hz, -CH(H)-His); 2.86 (d, 1H, J = 5.0 Hz, -CH(H)-His); 2.95 (s, 9H, CH_3-HDTMA); 3.12 (t, 2H, J = 8.4 Hz, -CH_2-HDTMA); 3.41 (t, 1H, J = 6.5 Hz, -CH-His); 6.76 (s, 1H, -CH(H)-His); 7.51 (s, 1H, -CH(H)-His); ^{13}C NMR (100 MHz, D_2O) δ, ppm: 13.88 (CH_3-HDTMA); 22.61 (-CH_2-HDTMA); 26.00 (-CH_2-HDTMA); 29.04 (-CH_2-HDTMA); 29.43 (-CH_2-HDTMA); 29.48 (-CH_2-HDTMA); 29.65 (-CH_2-HDTMA); 29.75 (-CH_2-HDTMA); 29.83 (-CH_2-HDTMA); 29.85 (-CH_2-HDTMA); 31.36 (-CH_2-His); 31.93 (-CH_2-HDTMA); 52.77 (-CH-His); 52.86 (-CH_3 HDTMA); 66.52 (-CH_2-HDTMA); 117.98 (-CH_2-His); 133.01 (-CH-His); 135.77 (-CH_2-His); 180.36 (-COO^- His); FT-IR ν_{max} (KBr, thin film): 3650-3000 (br), 2917, 2848, 1633, 1570, 1486, 1462, 1417, 964, 836, 730, 624 cm^{-1};

Hexadecylltrimethylammonium L-argininate ([HDTMA][L-Arg]):

^{1}H NMR (400 MHz, D_2O) δ, ppm: 0.93 (t, 3H, J = 6.9 Hz, CH_3-HDTMA); 1.28-1.43 (m, 26H, -CH_2-HDTMA); 1.61-1.66 (m, 4H, -CH_2-Arg); 1.75-1.84 (m, 2H, -CH_2-HDTMA); 3.16 (s, 9H, CH_3-HDTMA); 3.22 (t, 2H, J = 6.6 Hz, -CH_2-HDTMA); 3.27 (t, 1H, J = 5.0 Hz, -CH-Arg); 3.33 (t, 2H, J = 8.6 Hz, -CH_2-Arg); ^{13}C NMR (100 MHz, D_2O) δ, ppm: 13.86 (CH_3-HDTMA); 22.63 (-CH_2-HDTMA); 24.44 (-CH_2-HDTMA); 26.02 (-CH_2-HDTMA); 28.12 (-CH_2-Arg); 29.07 (-CH_2-

HDTMA); 29.47 (-CH_2-HDTMA); 29.52 (-CH_2-HDTMA); 29.70 (-CH_2-HDTMA); 29.88 (-CH_2-HDTMA); 29.91 (-CH_2-HDTMA); 31.60 (-CH_2-Arg); 31.96 (-CH_2-HDTMA); 40.93 (-CH_2-Arg); 52.81 (-CH_3 HDTMA); 55.53 (-CH-Arg); 66.50 (-CH_2-HDTMA); 156.86 (-CH-Arg); 183.18 (-COO^- Arg); FT-IR ν_{max} (KBr, thin film): 3600-3000 (br), 2917, 2850, 1647, 1570, 1507, 1487, 1462, 1407, 1330, 961, 730 cm^{-1};

Octadecylltrimethylammonium L-valinate ([HDTMA][L-Val]):

^1H NMR (400 MHz, D_2O) δ, ppm: 0.73-0.76 (d, 3H, CH_3-Val, t, 3H, CH_3-ODTMA); 0.82 (d, 3H, J = 6.8 Hz, CH_3-Val); 1.12-1.26 (m, 30H, -CH_2-ODTMA); 1.55-1.69 (m, 2H, -CH_2-ODTMA); 1.83-1.87 (m, 1H, -CH-Val); 2.97-3.00 (s, 9H, CH_3-ODTMA, m, 1H, -CH-Val); 3,18 (t, 2H, J = 8.6 Hz, -CH_2-ODTMA,); ^{13}C NMR (100 MHz, D_2O) δ, ppm: 13.86 (CH_3-ODTMA); 16.88 (-CH_3 Val); 19.11 (-CH_3 Val); 22.66 (-CH_2-ODTMA); 22.70 (-CH_2-ODTMA); 26.14 (-CH_2-ODTMA); 29.23 (-CH_2-ODTMA); 29.53 (-CH_2-ODTMA); 29.71 (-CH_2-ODTMA); 29.86 (-CH_2-ODTMA); 29.96 (-CH_2-ODTMA); 30.00 (-CH_2-ODTMA); 30.03 (-CH_2-ODTMA); 30.06 (-CH_2-ODTMA); 31.43 (-CH-Val); 32.00 (-CH_2-ODTMA); 52.82 (-CH_3 ODTMA); 61.66 (-CH-Val); 66.42 (-CH_2-ODTMA); 180.59 (-COO^-Val); FT-IR ν_{max} (KBr, thin film): 3600-3000 (br), 2918, 2849, 1669, 1569, 1473, 1463, 1408, 1296, 1165, 965, 912, 719 cm^{-1};

Octadecylltrimethylammonium L-methioninate ([HDTMA][L-Met]):

^1H NMR (400 MHz, D_2O) δ, ppm: 0.76 (t, 3H, J = 7.0 Hz, CH_3-ODTMA); 1.11-1.28 (m, 30H, -CH_2-ODTMA); 1.62-1.67 (m, 2H, -CH_2-ODTMA, m, 1H, CH(H)-Met); 1.80-1.86 (m, 1H, CH(H)-Met); 1.97 (s, 3H, CH_3-Met); 2.44 (t, 2H, J = 8.3 Hz, -CH_2-Met); 3.00 (s, 9H, CH_3-ODTMA); 3.20 (t, 2H, -CH_2-ODTMA, t, 1H, -CH-Met); ^{13}C NMR (100 MHz, D_2O) δ, ppm: 13.87 (CH_3-ODTMA); 14.31 (-CH_3 Met); 22.66 (-CH_2-ODTMA); 22.73 (-CH_2-ODTMA); 26.17 (-CH_2-ODTMA); 29.24 (-CH_2-ODTMA); 29.51 (-CH_2-ODTMA); 29.71 (-CH_2-ODTMA); 29.84 (-CH_2-ODTMA); 29.94 (-CH_2-ODTMA); 29.97 (-CH_2-ODTMA); 30.02 (-CH_2-Met); 31.99 (-CH_2-ODTMA); 34.11 (-CH_2-Met); 52.85 (-CH_3 ODTMA); 55.24 (-CH-Met); 66.48 (-CH_2-ODTMA); 181.12 (-COO^- Met); FT-IR ν_{max} (KBr, thin film): 3700-3000 (br), 2919, 2850, 1582, 1509, 1487, 1473, 1463, 1407, 1317, 719 cm^{-1};

Octadecylltrimethylammonium L-histidinate ([HDTMA][L-His]):

^{1}H NMR (400 MHz. D_2O) δ. ppm: 0.74 (t. 3H. J = 6.7 Hz. CH_3-ODTMA); 1.05-1.23 (m. 30H. -CH_2-ODTMA); 1.50-1.64 (m. 2H. -CH_2-ODTMA); 2.71 (d. 1H. J = 7.9 Hz. -CH(H)-His); 2.87 (d. 1H. J = 5.2 Hz. -CH(H)-His); 2.94 (s. 9H. CH_3-ODTMA); 3.12 (t. 2H. J = 8.5 Hz. -CH_2-ODTMA); 3.43 (t. 1H. J = 5.1 Hz. -CH-His); 6.77 (s. 1H. -CH(H)-His); 7.50 (s. 1H. -CH(H)-His); ^{13}C NMR (100 MHz. D_2O) δ. ppm: 13.89 (CH_3-ODTMA); 22.65 (-CH_2-ODTMA); 26.08 (-CH_2-ODTMA); 29.17 (-CH_2-ODTMA); 29.50 (-CH_2-ODTMA); 29.62 (-CH_2-ODTMA); 29.79 (-CH_2-ODTMA); 29.83 (-CH_2-ODTMA); 29.92 (-CH_2-ODTMA); 29.96 (-CH_2-ODTMA); 30.01 (-CH_2-ODTMA); 31.30 (-CH_2-His); 31.98 (-CH_2-ODTMA); 52.77 (-CH-His); 52.82 (-CH_3 ODTMA); 66.50 (-CH_2-ODTMA); 117.95 (-CH_2-His); 132.99 (-CH-His); 135.76 (-CH_2-His); 180.08 (-COO^- His); FT-IR ν_{max} (KBr, thin film): 3650-3000 (br), 2918, 2850, 1634, 1578, 1463, 1342, 1315, 1250, 966, 912, 834, 720, 624 cm^{-1};

Octadecylltrimethylammonium L-argininate ([HDTMA][L-Arg]):

^{1}H NMR (400 MHz. D_2O) δ. ppm: 0.80 (t, 3H, J = 7.0 Hz, CH_3-ODTMA); 1.17-1.30 (m, 30H, -CH_2-ODTMA); 1.49-1.52 (m, 4H, -CH_2-Arg); 1.64-1.70 (m, 2H, -CH_2-ODTMA); 3.04 (s, 9H, CH_3-ODTMA); 3.10 (t, 2H, J = 7.5 Hz, -CH_2-Arg); 3.15 (t, 1H, J = 5.1 Hz, -CH-Arg); 3.20 (t, 2H, J = 7.9 Hz, -CH_2-ODTMA); ^{13}C NMR (100 MHz, D_2O) δ, ppm: 13.87 (CH_3-ODTMA); 22.68 (-CH_2-ODTMA); 24.44 (-CH_2-ODTMA); 26.11 (-CH_2-ODTMA); 29.18 (-CH_2-Arg); 29.56 (-CH_2-ODTMA); 29.65 (-CH_2-ODTMA); 29.84 (-CH_2-ODTMA); 29.90 (-CH_2-ODTMA); 30.00 (-CH_2-ODTMA); 30.02 (-CH_2-ODTMA); 30.04 (-CH_2-ODTMA); 30.10 (-CH_2-ODTMA); 31.55 (-CH_2-Arg); 32.03 (-CH_2-ODTMA); 40.95 (-CH_2-Arg); 52.87 (-CH_3 ODTMA); 55.53 (-CH-Arg); 66.51(-CH_2-ODTMA); 156.74 (-CH-Arg); 183.05 (-COO^- Arg); FT-IR ν_{max} (KBr. thin film): 3600-3000 (br), 2918, 2849, 1669, 1569, 1473, 1463, 1408, 1296, 1165, 965, 924, 719 cm^{-1};
